# Neuromechanistic Model of Auditory Bistability

**DOI:** 10.1371/journal.pcbi.1004555

**Published:** 2015-11-12

**Authors:** James Rankin, Elyse Sussman, John Rinzel

**Affiliations:** 1 Center for Neural Science, New York University, New York, New York, United States of America; 2 Dominick P. Purpura Department of Neuroscience, Albert Einstein College of Medicine, Bronx, New York, United States of America; 3 Department of Otorhinolaryngology-HNS, Albert Einstein College of Medicine, Bronx, New York, United States of America; 4 Courant Institute of Mathematical Sciences, New York University, New York, New York, United States of America; Johns Hopkins University, UNITED STATES

## Abstract

Sequences of higher frequency A and lower frequency B tones repeating in an ABA- triplet pattern are widely used to study auditory streaming. One may experience either an integrated percept, a single ABA-ABA- stream, or a segregated percept, separate but simultaneous streams A-A-A-A- and -B---B--. During minutes-long presentations, subjects may report irregular alternations between these interpretations. We combine neuromechanistic modeling and psychoacoustic experiments to study these persistent alternations and to characterize the effects of manipulating stimulus parameters. Unlike many phenomenological models with abstract, percept-specific competition and fixed inputs, our network model comprises neuronal units with sensory feature dependent inputs that mimic the pulsatile-like A1 responses to tones in the ABA- triplets. It embodies a neuronal computation for percept competition thought to occur beyond primary auditory cortex (A1). Mutual inhibition, adaptation and noise are implemented. We include slow NDMA recurrent excitation for local temporal memory that enables linkage across sound gaps from one triplet to the next. Percepts in our model are identified in the firing patterns of the neuronal units. We predict with the model that manipulations of the frequency difference between tones A and B should affect the dominance durations of the stronger percept, the one dominant a larger fraction of time, more than those of the weaker percept—a property that has been previously established and generalized across several visual bistable paradigms. We confirm the qualitative prediction with our psychoacoustic experiments and use the behavioral data to further constrain and improve the model, achieving quantitative agreement between experimental and modeling results. Our work and model provide a platform that can be extended to consider other stimulus conditions, including the effects of context and volition.

## Author Summary

Humans have an astonishing ability to separate out different sound sources in a busy room: think of how we can hear individual voices in a bustling coffee shop. Rather than voices, we use sound stimuli in the lab: repeating patterns of high and low tones. The tone sequences are ambiguous and can be interpreted in different ways—either grouped into a single stream, or separated out into different streams. When listening for a long time, one’s perception switches every few seconds, a phenomenon called auditory bistability. Based on knowledge of the organization of brain areas involved in separating out different sound sources and how neurons in these areas respond to the ambiguous sequences, we developed a computational model of auditory bistabilty. Our model is less abstract than existing models and shows how groups of neurons may compete in order to dictate what you perceive. We predict how the difference between the two tone sequences affects what you hear over time and we performed an experiment with human listeners to confirm our prediction. The model provides groundwork to further explore the way the brain deals with the busy and often ambiguous world of sound.

## Introduction

**Fig 1:**
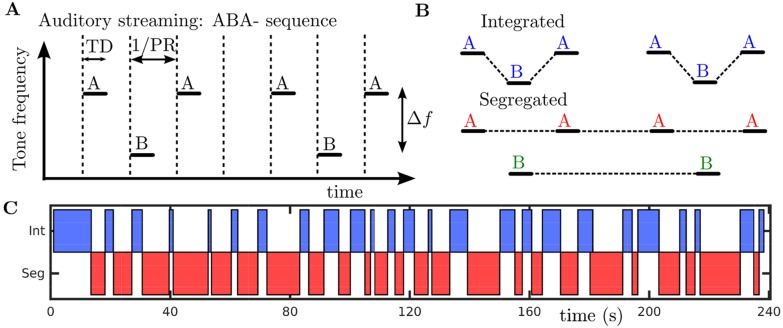
Stimulus paradigm and two possible percepts. **A**: Repeating ABA- triplet sequences (two triplets shown) consist of higher frequency pure tones A interleaved with lower frequency pure tones B of duration *TD* separated by a frequency difference Δ*f*. The time between tone onsets (dashed vertical lines) is inverse of the presentation rate 1/*PR* (the “-” in “ABA-” represents a silence of duration 1/*PR*). Throughout this paper tone duration will be set to *TD* = 1/*PR* such that offset of an A tone abuts the onset of the next B tone. **B**: The stimulus is perceived as either integrated into a single stream ABA-ABA- or as two separate streams A-A-A-A- and -B---B--. **C**: Subject reports of integrated and segregated from a single 4-minute trial (480 triplets) at Δ*f* = 5 st and *PR* = 8 Hz.

Auditory scene analysis involves segregating a complex scene into individual objects or streams [1]. A common stimulus used to study streaming in psychoacoustic experiments involves alternating tone sequences organized in repeating ABA- triplets [2]. The sequence can be perceived integrated in to one, or segregated into two streams, see Fig 1A and 1B. A key point of comparison in studies of streaming has been the van Noorden diagram, which describes the predominance of different interpretations across ranges of two stimulus parameters: the difference (Δ*f*) in frequency between the A and the B tones and the tone presentation rate (PR). The diagram describes parameter regions where integrated is predominant (smaller Δ*f*), where segregated is predominant (larger Δ*f*), or where perception is ambiguous between the two (an intermediate range, also dependent on *PR*). Over a large range of stimulus parameter values the initial percept is integrated, but as time proceeds a perceptual switch to the segregated interpretation becomes more likely, a phenomenon called the build up of stream segregation [1, 3, 4]. At intermediate values of Δ*f* the build up to segregation takes a few to ten seconds, but can take extend to tens of seconds at small Δ*f* and occur almost instantaneously at large Δ*f*. Behavioral studies have also looked at the effects of attention [5–7] and context [8–10] for build up.

Several computational models have focused on the early phase of auditory streaming, reproducing some characteristic features of build up [11–13], of the van Noorden diagram [14–16] or of both [17]. Various modelling approaches have been employed to reach these goals, including a coupled oscillator networks with frequency and time dimensions [15], a neural field description with a continuous representation of tonotopy [16] and a statistical model treating build up as an alternating renewal process [13]. The models are typically quite abstract, although some include a hybrid of auditory pathway qualitative features and signal processing [11, 14, 17]. However, these models were not neuromechanistic in nature; they did not account for neuronal dynamics based on explicit neurophysiological mechanisms nor did they study dynamics beyond a first perceptual switch and the build up phase.

During long stimulus presentations, on the order of several minutes, the initial switch to segregated is typically followed by further switches back and forth between segregation and integration [18–21], as illustrated by a subject’s reporting of current percept in Fig 1C. Imaging studies have shown activation of a thalamocortical network [22] and the cerebellum [23] around the time of perceptual switches and an MEG study [18] localised to auditory cortex implicates non-primary auditory areas in maintaining perceptual streams. Psychoacoustic studies have shown that the initial perceptual phase is typically longer than the subsequent percept durations [21] and that mean percept durations remain stable from the second perceptual phase onward [19]. The phenomenological CHAINS model presented in [24] is an implementation of the two stage proposal outlined in [25], addressing algorithmically the formation of the different perceptual patterns that enter into competition and capturing the later competition between these organizations. Aside from the CHAINS model [24], the available auditory modelling literature has not addressed the persistent perceptual alternations following the build up phase for the auditory streaming paradigm. The persistent, irregular alternations after the first perceptual switch will be the focus of this study.

Numerous neurophysiology studies using short stimulus presentations (say, less than 20 s) are of particular relevance to our modeling approach. Trial averaged recordings show a dependence on Δ*f* and *PR* in the primary auditory cortex (A1) of awake monkeys [26–28], in the forebrain of songbirds [29] and in the cochlear nucleus of anaesthetized guinea pigs [30]. A1 responses were interpreted in a statistical model using neurometric functions that mimic the time course of build up in [28, 30]. In other studies data have been interpreted according to a conceptual model [26, 29] based on the tonotopic organization in auditory cortex. When the A and B tones are far enough apart in frequency, cortical activity is sufficiently separated in tonotopic space to drive segregated representations of the high and low tone sequences. However, when the A and B tones are closer, there is enough receptive field overlap for both tones to drive a common, intermediate population that could encode the integrated percept. There is no evidence (nor do the authors conclude) that the neural substrate for the first perceptual switch is to be found in A1 and further experiments in the behaving songbird suggest stimulus features, but not perceptual choice, are encoded in A1 [31].

In this study we develop a neuromechanistic model of auditory streaming that focuses on accurately reproducing the dynamics of the alternations after the build up phase (i.e. after the first perceptual switch). Our formulation is directly motivated from physiological studies of auditory streaming [26, 28] and general models of perceptual bistability [32–34]. A reduction from a continuous feature representation to percept based inputs and competition was proposed in [32], and this approach has become relatively standard in general models of perceptual rivalry. By contrast, input in our model directly represents the dynamics of sensory features and incorporates neuronal responses of pre-competition stages. It features a tonotopic organization with three units assumed to be downstream and receive input from A1. Two peripheral units receive input from regions of A1 centred at locations with best frequencies A or B, and a third unit receives input from tonotopically intermediate A1 locations, say centred at (A + B)/2. The perceptual interpretations are classified through criteria on the firing patterns, for example, dominance of the central unit, receiving input via A1 from both the A and the B tones, corresponds to integrated [26]. A dynamical systems framework is used with firing rate based neuronal competition mediated by mutual inhibition, adaptation and noise [32, 34]; this combination of neural mechanisms has proved successful in accounting for many of the characteristic behaviors of perceptual bistability. Our model incorporates in its inputs the onset and transient dynamics of A1 responses with gaps between triplets. The inclusion of recurrent NMDA-like excitation allows for some neuronal memory that links each tone and each triplet to the next.

A starting hypothesis is that the perceptual organization known from [2] for initial dominance phases extends to long presentations; this allows for the tonotopic distribution of input and lateral connectivity to be constrained. As explained in more detail in the *Discussion*, comparisons with van Noorden’s experimental findings are frequently made in experiments and models that do not take into account time-varying stimuli, attention and the termination of trials at the first perceptual switch. Accordingly, the starting assumption with regards to the van Noorden organization is re-evaluated based our own experimental findings. A further point of comparison from the empirical literature will be the log-normal- or gamma-like distribution of dominance durations characteristic of perceptual bistability [35]. Two existing auditory studies have reported durations resembling the characteristic distributions [19, 24]. Here, we will use the dominance duration distributions from our own experiments outlined below to constrain model parameters.

In parallel and interacting with the model development we have established in psychoacoustic experiments how parameter manipulations (here Δ*f*) affect the dominance of competing percepts. Importantly, we have confirmed that a generalization about parameter manipulations from visual bistability extends to the auditory case. Levelt’s Proposition II (LPII) described the specific way that asymmetric contrast manipulations in binocular rivalry can shift the proportion of dominance from one perceptual interpretation to another [36, 37]. In [38], LPII was generalized for three bistable visual stimuli varying parameters either side of equidominance. Equidominance coincides with a choice of stimulus parameters where each of the two interpretations are dominant for an equal proportion of time. It was shown that as a parameter is varied, the durations of the stronger percept (dominant for a larger proportion of time) are affected more than the durations of the weaker percept. Although some existing studies recorded perceptual durations for a similar stimulus paradigm (with minutes-long trials), data was not been presented so as to allow for a comparison with gLPII. For example, [21] considered measures that did not separate the two percept types and [24] did not separate the first phase from subsequent phases in their analysis. Our experiment also provides a means to test model predictions, to constrain its parameters and to evaluate its underlying assumptions. Indeed, the model was developed concurrently with the design and execution of the experiments, a process that has allowed us to develop a deeper understanding as one discipline feeds into and informs the other. The results in this paper are organized to reflect this process. The model is described in detail in *Neuromechanistic model of auditory bistability*, initial predictions are described in *Parametric dependence of perceptual dominance: model*, experimental results are in *Parametric dependence of perceptual dominance: experiment*, and a comparison with the model is presented in and *Refined model better captures experimental results*.

## Results

### Neuromechanistic model of auditory bistability

The neuronal circuits for competition and perceptual encoding are assumed to be downstream and taking inputs from A1 (see the Discussion, specifically *Model of neuronal competition beyond A1*, for comments on the possible cortical location of our model). The periodic inputs mimic the A1-responses to ABA- sequences reported in [28]. A firing rate description is used where competitive interactions emerge through a combination of excitatory and inhibitory connections, a slow fatigue process and intrinsic noise. We provide a brief outline of the model’s architecture, mechanisms and inputs here; the full model equations and further details are given in *Model equations and details*.

**Fig 2:**
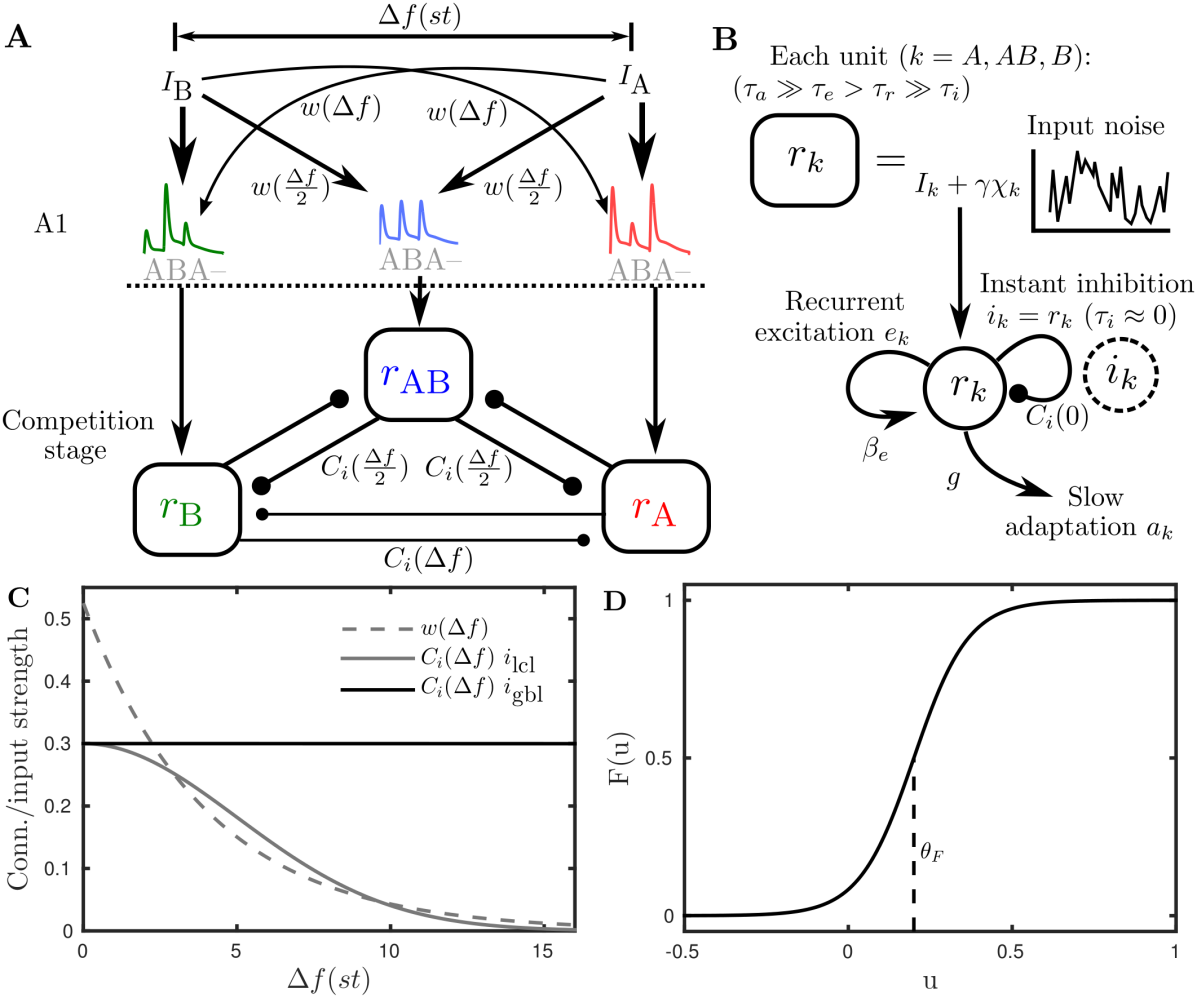
Model architecture. **A**: The tone frequency difference between A and B is Δ*f*. The spread of inputs *I*_A_ and *I*_B_ across A1 is governed by the decaying input gain function *w*(Δ*f*). Example A1 response patterns to the ABA- stimulus are shown (see Fig 3 and associated text for more details); these form the inputs to three neuronal populations *r*_A_, *r*_B_ and *r*_AB_ at the competition stage. Lateral inhibition strength can depend on Δ*f* (*i*_lcl_ case) or be independent of Δ*f* (*i*_gbl_ case) as governed by *C*_*i*_(Δ*f*). **B**: Each population has a slow adaptation *a*_*k*_ on a timescale *τ*_*a*_ with strength *g*, recurrent excitation *e*_*k*_ on an intermediate timescale *τ*_e_ with strength *β*_*e*_ and an independent noise source *χ*_*k*_ with strength *γ*. Slow synaptic depression *d*_*k*_ on the recurrent excitation *e*_*k*_ is not shown. Recurrent inhibition *i*_*k*_ with strength *C*_*i*_(0) is instantaneous allowing for the simplification *i*_*k*_ = *r*_*k*_. See Eq (1) for an illustrative single-unit equation and *Model equations and details* for the full model (Eq (4)). **C**: The Δ*f*-dependent profiles for the input spread *w*(Δ*f*) (exponential decay (Eq (7))) and lateral inhibition *C*_*i*_(Δ*f*) (Gaussian decay (Eq (6)) for *i*_lcl_ or constant *β*_*i*_ for *i*_gbl_). **D**: Sigmoidal firing rate function *F*(*u*) (Eq (5)) with maximal slope *k*_*F*_/4 at the threshold *θ*_*F*_.

The schematic in Fig 2A shows units *r*_A_, *r*_B_ and *r*_AB_ that respectively pool inputs from regions of A1 centred at locations with best frequencies A, B and somewhere between, say (A + B)/2. The frequency difference between A and B is Δ*f*. The associated variables *r*_*k*_ represent the mean firing rate of a population of neurons centred at the corresponding tonotopic location *k* (*k* = {*A*, *AB*, *B*}). The inputs *I*_A_ and *I*_B_, described in more detail below, are distributed between the units (arrows) with less of the input *I*_B_ (*I*_A_) reaching *r*_AB_ and *r*_A_ (*r*_B_) with increasing Δ*f* as controlled by *w*(Δ*f*) (plotted in panel C). The amount of mutual inhibition (circular-terminated vertices) between the units also depends on Δ*f* as controlled by *C*_*i*_(Δ*f*) (plotted in panel C for local *i*_lcl_ and global *i*_gbl_ inhibition cases, see below). For each unit *r*_*k*_ the intrinsic dynamics are illustrated in panel B and decribed by a differential equation like the following,

$$\begin{array}{ll} \tau_{r}{\dot{r}}_{\text{AB}}= & -r_{\text{AB}}+F(\beta_{e}d_{\text{AB}}e_{\text{AB}}\\ & -C_{i}\left(0\right)r_{\text{AB}}-C_{i}\left(\Delta f/2\right)\left(r_{A}+r_{B}\right)-ga_{\text{AB}}+w\left(\Delta f/2\right)\left(I_{A}+I_{B}\right)+\chi_{\text{AB}}). \end{array}$$ (1)

By way of an example, we describe this equation for *r*_AB_ in detail; the equations for *r*_A_ and *r*_B_ take the same general form. The dynamics evolve on a cortical timescale *τ*_*r*_ with a decay term and a sigmoidal function *F* that transforms all local and lateral inputs to a firing rate, see Fig 2D. Recurrent excitation has strength *β*_*e*_ and the excitation variable *e*_AB_ evolves on a timescale *τ*_e_, slower than the cortical dynamics. The inclusion of slow NMDA-like excitation was necessary in order to maintain activity during the silent phases between successive inputs. The recurrent excitation term also features slow synaptic depression *d*_AB_ operating on a timescale *τ*_*d*_. The function *C*_*i*_(Δ*f*) controls the strength of local inhibition at Δ*f* = 0 and lateral inhibition from *r*_A_ and *r*_B_, a distance $\frac{\Delta f}{2}$ away. Net local excitation is ensured by setting *β*_*e*_ > *C*_*i*_(0) = *β*_*i*_. A simplification, that inhibition is instantaneous *τ*_*i*_ ≈ 0, removes the need for an explicit inhibition variable *i*_*k*_ and inhibition is proportional to the cortical variables *r*_*k*_. Simulations were run (not shown) to verify that this simplification did not have a significant effect on the model’s dynamics. Intrinsic spike frequency adaptation has strength *g* and the adaptation variable *a*_AB_ evolves on a slow timescale *τ*_*a*_. Intrinsic additive noise *χ*_AB_ is an independent Ornstien-Uhlenbeck process for each *r*_*k*_.

**Fig 3:**
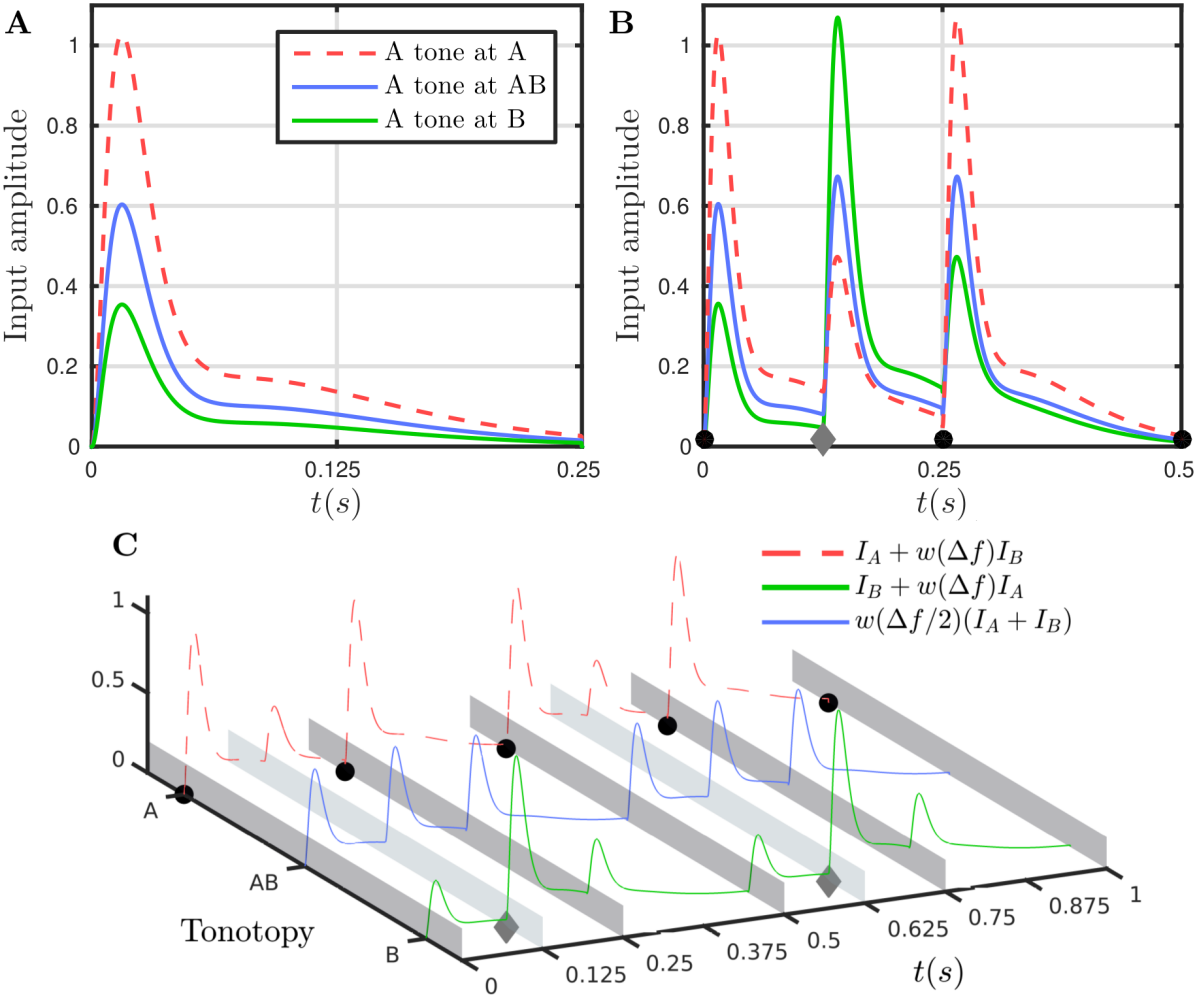
Model inputs. **A**: Input time courses are represented by double alpha functions (see *Model equations and details* and Eq (8)) that capture the onset and plateau characteristics of A1-responses from [28]. For a single 125 ms tone of frequency A less input will arrive at locations AB and B than at A as described by Eq (7) and plotted here for Δ*f* = 4 [26]. **B**: Inputs (see legend in C) to the respective populations *r*_A_, *r*_B_ and *r*_AB_ for an ABA- triplet of 0.5 s (tone duration and post-triplet silence “-” of 125 ms, i.e. *PR* = 8 Hz). Tone onsets: black circle for A-tone, gray diamond for B-tone. **C**: As B with curves distributed across the model’s tonotopy. The A-tone input is full amplitude at the A location, less at the AB location and further less at the B location, correspondingly for the B-tone input.

Inputs to the model are illustrated in Fig 3. The onset-plateau shape of A1 responses to a single tone mimic [28] and are shown in panel A for an isolated 125 ms A tone at locations A, AB and B with Δ*f* = 4 st. The inputs are illustrated here for a presentation rate (inverse of the tone durations) of *PR* = 8 Hz (= 1/125); no gaps are assumed between the tones (*TD* = 1/*PR*) and tone durations are assumed vary with PR accordingly. The input spread across tonotopy is mediated by *w*(Δ*f*) as plotted in Fig 2C. The overall input for an ABA- triplet at the three locations is illustrated in panel B. The same curves are plotted for two triplets distributed across the tonotopic axis in panel C. Note that there is a full response to the A tone at the location A, an intermediate responses to both tones at the location AB and a full response to the B tones at the location B.

In the first part of the modeling results the slow synaptic depression variable *d*_AB_ is frozen at 1 and inhibition is localized in Δ*f* (see corresponding *C*_*i*_(Δ*f*) curve *i*_lcl_ in Fig 2C). The symbols *e*_fix_ and *i*_lcl_ are used to make these mechanistic distinctions. In the second part of the modeling results there is a dynamic slow decay *d*_AB_ on the excitation and inhibition is global in Δ*f* (see corresponding *C*_*i*_(Δ*f*) curve *i*_gbl_ in Fig 2C). The symbols *e*_dyn_ and *i*_gbl_ are used to make these mechanistic distinctions. A comparison between the cases with (*e*_fix_, *i*_lcl_) and (*e*_dyn_, *i*_gbl_) is made in *Refined model better captures experimental results*.

The full set of fifteen model equations (three principal equations like Eq (1) and twelve simple equations for the *e*_*k*_-, *a*_*k*_-, *d*_*k*_- and *χ*_*k*_-variables) are given in *Model equations and details* along with the full expressions for *F*, *w*(Δ*f*), *C*_*i*_(Δ*f*), *I*_A_ and *I*_B_. All model parameter values are given in Table 1.

**Table 1:** Model parameters as defined in *Neuromechanistic model of auditory bistability* and forming part of the general model equations given in Eq (4).

Parameter	Description	Model (*e*_fix_, *i*_lcl_)	Model (*e*_dyn_, *i*_gbl_)
Firing rate:			
*θ*_*F*_	Sigmoid threshold	0.2	unchanged
*k*_*F*_	Sigmoid slope	12	unchanged
Input:			
Λ_2_	Input plateau fraction	1/6	unchanged
*α*_1_	Input impulse rise time	15 ms	unchanged
*α*_2_	Input plateau stay time	82.5 ms	unchanged
*I*_*p*_	Input amplitude	0.525	0.47
*σ*_*p*_	Lateral input decay	8 st	8.5 st
Intrinsic:			
*g*	Adaptation strength	0.065	unchanged
*γ*	Noise strength	0.075	unchanged
*β*_*i*_	Inhibition strength	0.3	unchanged
*σ*_*i*_	Inhibition decay constant	10 st	*σ*_*i*_ → ∞ (global)
*β*_*e*_	Recurrent excitation strength	0.7	0.85
*κ*	Excitation decay strength	0	0.25
Timescales:			
*τ*_*r*_	Cortical timescale	10 ms	unchanged
*τ*_*a*_	Adaptation timescale	1.4 s	unchanged
*τ*_e_	NMDA excitation timescale	70 ms	unchanged
*τ*_X_	Noise timescale	100 ms	unchanged
*τ*_*d*_	Excitation decay timescale	N/A	3 s

### Encoding of percepts and dynamics of alternations: model

**Fig 4:**
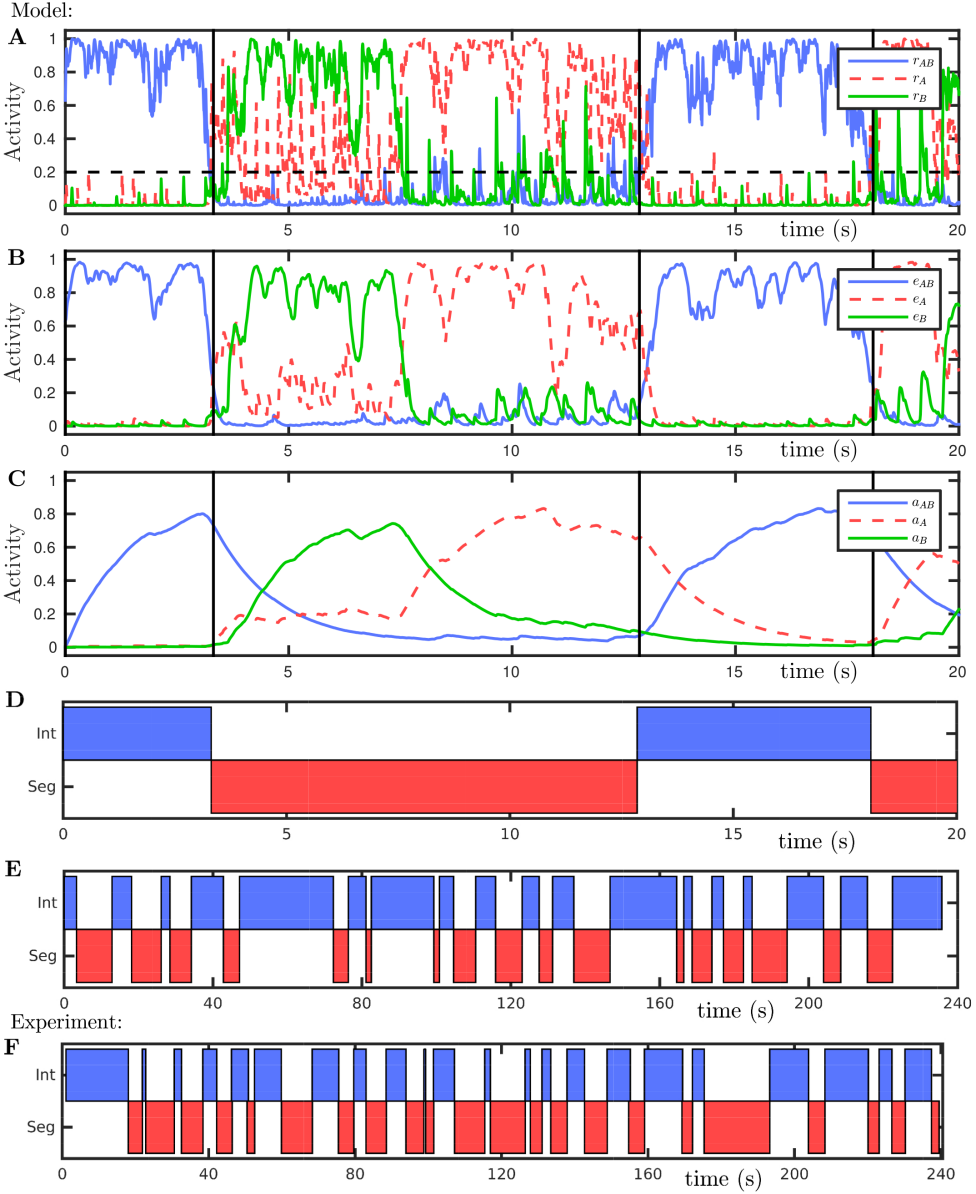
Time courses of model responses (A–C), predicted percepts (D–E), and an example of perceptual reports from our psychoacoustic experiments (F). **A**: Population firing rate time courses with Δ*f* = 5 st and *PR* = 8 Hz. The firing rate function threshold *θ*_*F*_ is a horizontal dashed black line. Abrupt perceptual switches are seen when AB firing decreases/increases drastically (vertical black lines); see text for the exact criterion for a switch. **B**: As in A, here for the synaptic excitation variables. **C**: As in A for the adaptation variables. **D**: Percept as encoded from A, see text. **E**: Encoded percept for the full 240 s simulation; panels A–D show only first 20 s. **F**: Time course of continuous percept reporting in psychoacoustic experimental for a 240 s trial at Δ*f* = 5 st.

We first discuss the output from individual simulations of the model and illustrate how the model’s firing rate variables can encode the competing percepts for comparison with experimental data. Fig 4 shows time histories for a model simulation, panels A–D represent the first 20 s of a 240 s simulation at Δ*f* = 5 st and *PR* = 8. These stimulus parameters correspond to the ambiguous region where perception is bistable and regular alternations take place [19]. Panel A shows the primary population’s variables evolving on the cortical timescale *τ*_*r*_. When central unit *r*_AB_ is active (e.g. during the first 3 s), the peripheral units *r*_A_ and *r*_B_ are suppressed. During this time the adaptation *a*_AB_ builds up until the peripheral units become active, see panel C. Conversely, when either or both of the peripheral units *r*_A_ and *r*_B_ are active, the central unit *r*_AB_ is suppressed. When *r*_AB_ is suppressed, typically one of *r*_A_ or *r*_B_ will be more active, reflecting the fact that either the A tones or the B tones are in the foreground during the segregated percept. The possibility of considering perception as tristable or multistable is discussed further in the discussion. The NMDA-like excitation variables, evolving on a slower timescale *τ*_e_ than the cortical timescale *τ*_*r*_, shown in panel B are effectively a temporal smoothing of the cortical variables in panel A. Note that the response transients for individual inputs seen in panel A (look at the sub-threshold activity) are integrated across several inputs in panel B (seen most clearly around 10 s). In our model, activation of the peripheral units encodes the segregated percept, while activation of the central unit encodes the integrated percept. This classification of integrated relies on an implicit assumption of a stimulus featuring a combination of A and B tones where activation of the central unit reflects a linking of the two sequences. An AND-like computation (e.g. classify integrated as when As and Bs are present AND central unit is active) could be implemented to distinguish between an ABA- stimulus and an unambiguous sequence CCC- where C = (A + B)/2. Moreover, the CCC- input should not be confused with integration since the central unit would suppress the peripheral units indefinitley and no alternations would take place.

From a practical point of view, our criteria for classifying the integrated and segregated percepts are based, as follows, on the time courses of the model output plotted in Fig 4. Vertical black lines in panels A–C indicate switches in dominance between the central unit *r*_AB_ and the peripheral units *r*_A_ and *r*_B_. These switches can be computed algorithmically in the following way. First the output variables *r*_AB_, *r*_A_ and *r*_B_ are processed with a temporal moving average filter with 50 ms width to obtain smoothed readouts $\tilde{r_{\text{AB}}}$, $\tilde{r_{A}}$ and $\tilde{r_{B}}$, these look similar to the excitation variable traces shown in panel B. The output is encoded as integrated when $\tilde{r_{\text{AB}}}>\left(\tilde{r_{A}}+\tilde{r_{B}}\right)/2$ and segregated otherwise. The mutual inhibition between the populations gives rise to sharp well defined crossovers with steep gradient at points where $\tilde{r_{\text{AB}}}=\left(\tilde{r_{A}}+\tilde{r_{B}}\right)/2$. In this way the model output is encoded as integrated when *r*_AB_ is dominant and segregated when *r*_A_ and *r*_B_ are dominant, as plotted in panel D (first 20 s) and panel E (full 240 s). This allows for a direct comparison between the model simulations and perceptual reports from experiment, an example of which is shown in panel F; the experiment will be discussed in *Parametric dependence of perceptual dominance: experiment* and a full comparison with the model made in *Refined model better captures experimental results*.

### Statistics of dominance durations: model

Percept durations for perceptually bistable stimuli have been shown to be fit well by gamma or log-normal distributions, for example, see [39–41]. As has become standard in the analysis of percept durations for bistable stimuli, the switching times are normalized by the mean for each percept type (integrated or segregated) [40]. The coefficient of variation (CV), which is the ratio of the standard deviation *s* with the mean $\bar{x}$ ($\text{CV}=\frac{s}{\bar{x}}$), is used as a measure of variability in the percept durations, where smaller values indicate more adaptation-driven alternations, and larger values more noise-driven alternations [34]. During the design of the model some pilot data was used to constrain the model, but informality in its collection preclude its publication. Nevertheless, we do make a comparison with data from the final experiment as reported in detail *Parametric dependence of perceptual dominance: experiment*, which were collected after the development and tuning of model parameters.

**Fig 5:**
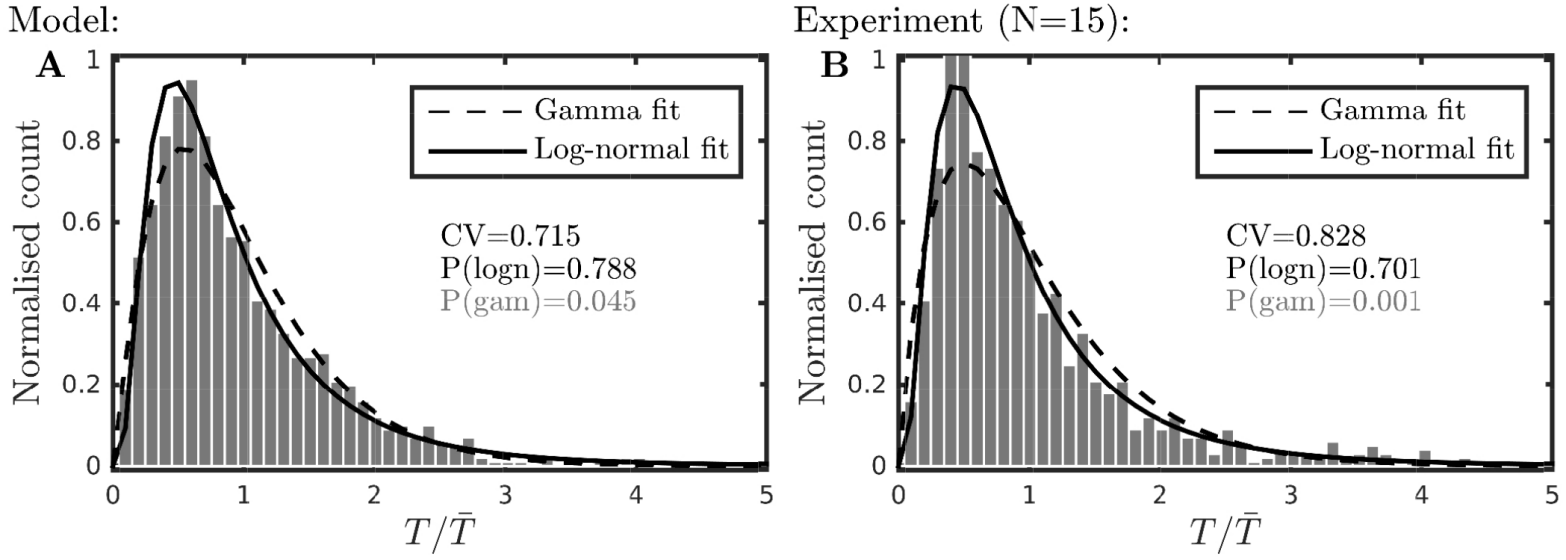
Statistics of dominance durations. **A**: Histogram of 1000 durations from model simulations at Δ*f* = 5 combined across perceptual type after normalising by the mean, see text. Curves show best-fit by gamma and log-normal distributions, P-values from one-way KS test are shown (in gray if the distribution can be rejected at the 0.05 significant level). **B**: As in A, here for the experimental condition Δ*f* = 5; normalized data combined across subjects.

In order to look at the distribution of perceptual durations in the model, 1000 durations were randomly sampled from 2225 durations computed from a total of 50 4-minute simulations at Δ*f* = 5 st and *PR* = 8 Hz. Model parameters controlling the balance between adaptation and noise were constrained to match pilot data collected from 6 subjects at Δ*f* = 5 and *PR* ≈ 8, which showed a mean duration of 4.3 s, a CV of 0.73 and a fit to a log-normal distribution (but not a gamma distribution). After parameter tuning, the model produced a mean duration of 5.1 s and the distribution has a CV of 0.72. These model results were consistent with our pilot data (the mean duration is longer but matching the CV was considered more important, given that durations are normalized for most of the analysis in this paper). Furthermore, model data showed a fit to a log-normal, but not a gamma distribution, see Fig 5A (details of the statistical tests for fits to standard distribution are given in *Comparison to standard statistical distributions*).

We make a comparison with the distributions from subsequent experiments (reported in full in *Parametric dependence of perceptual dominance: experiment*) where, similarly, 1000 durations were randomly sampled from 1575 durations collected across a total of 45 4-minute trials (3 repetitions with *N* = 15 subjects) at Δ*f* = 5 st and *PR* = 8 Hz. The mean duration in the experiment was 7.7s and the distribution has a CV of 0.83. The data is best fit by a log-normal distribution, but not by a gamma distribution, see Fig 5B. A similar CV and fit to log-normal but not gamma was obtained for the other Δ*f* conditions. A two-way KS test comparing the normalized distributions for the model and experiment (plotted in Fig 5A and 5B) showed that the data could be drawn from the same underlying distribution (*P* = 0.19). Although there is a difference of 0.11 in CV values, this statistical test shows that the model has captured the dynamics of alternations in the experiment accurately. In the literature, mean durations across subjects ranging from ≈ 4 s to ≈ 9 s were reported in [19] with equivalent stimulus parameters. To our knowledge, there are no existing studies reporting the coefficient of variation in auditory bistabilty, but in the literature on bistable visual perception values for CV have been reported ranging from 0.48 to 0.67 across several paradigms [42] (*N* = 5–11 subjects) and from = 0.55 to 0.70 in binocular rivalry [40] (*N* = 3 subjects). The large CV value (> 0.7) in our auditory bistability experiment suggests that alternations, although driven by a combination of adaptation and noise, are more noise driven than the visual paradigms for which the CV has been reported [40, 42].

### Perceptual organization for stimulus parameters: model

**Fig 6:**
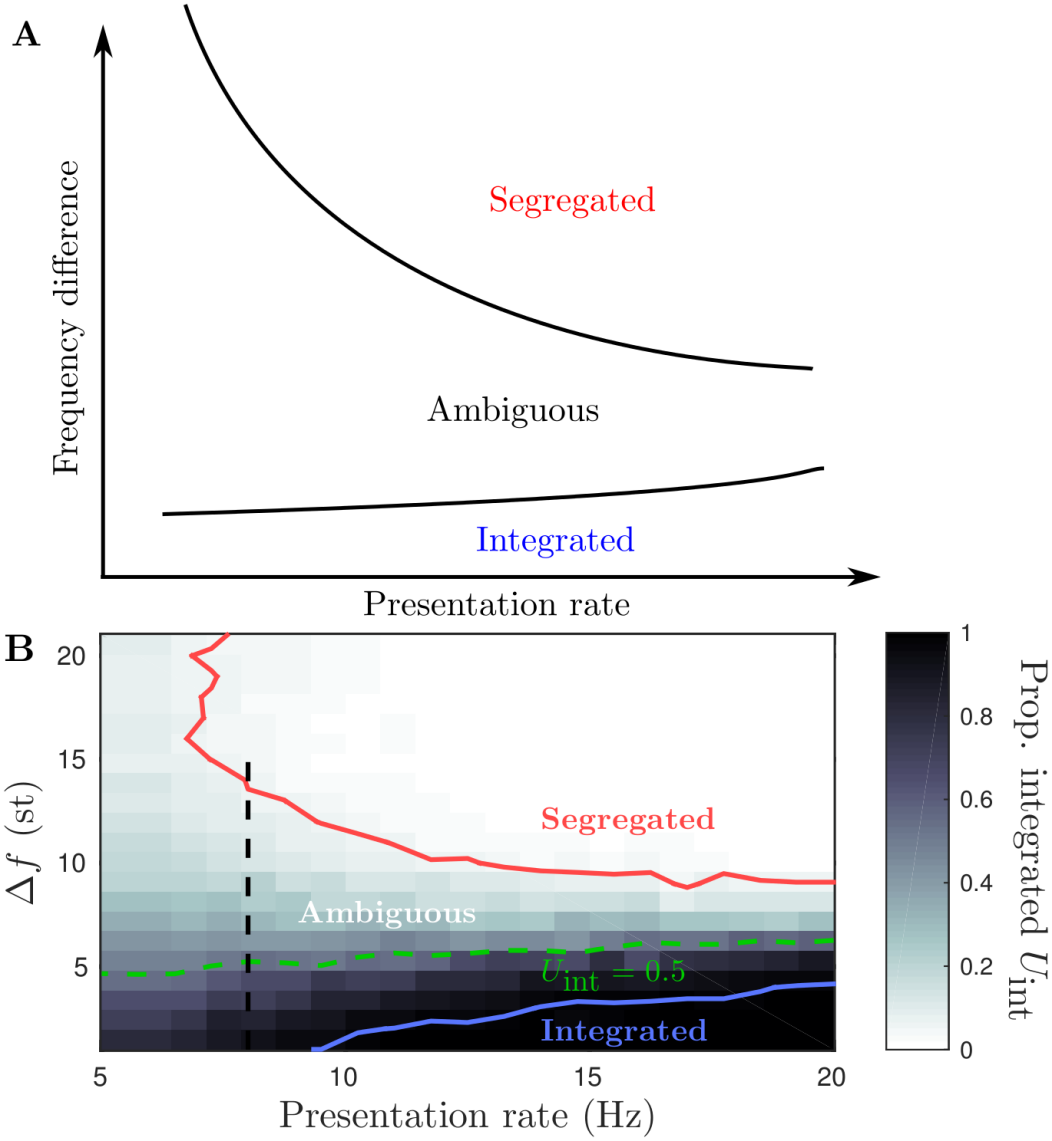
Perceptual organization for stimulus parameters. **A**: Schematic diagram of the perceptual regions in terms of presentation rate and frequency difference, redrawn after [2]. **B**: Grayscale map of proportion (of time) integrated *U*_int_ (see Eq (2)), segregated region is above red contour at *U*_int_ = 0.05, integrated region is below blue contour at *U*_int_ = 0.95, ambiguous region lies in between with equidominance at *U*_int_ = 0.5 along the dashed green contour. Vertical dashed line at *PR* = 8 corresponds to the frequency difference sweep used later in Figs 8 and 9.

The experiments in [2] used wide ranges of presentation rate *PR* and frequency difference Δ*f* to investigate how the predominance of the different interpretations can change. Three qualitative regions were mapped out in van Noorden’s experiments: at low Δ*f* integration is dominant, at large Δ*f* segregation is dominant and at intermediate Δ*f* perception is ambiguous. Fig 6A is a cartoon of the organization based upon [2]. Note that there is a further dependence of the boundaries between the regions on *PR*. The experiments in [2] focused only on the first transition and involved either dynamically varying stimuli or the deployment of the subject’s attention. The model we present specifically captures the dynamics of regular alternations after the initial transition, has a fixed stimulus and does not account for attention. Nevertheless, as a starting assumption, the same qualitative organization in terms of Δ*f* and *PR* is assumed for the model. Model parameters controlling the spread of input and lateral inhibitory connections, defined by the functions plotted in Fig 2C, were tuned to obtain the appropriate organization. In particular, it was necessary to include lateral inhibition *C*_*i*_(Δ*f*) that decays with Δ*f* as indicated by *i*_lcl_.

In order to define regions that are predominantly integrated, segregated or ambiguous we look at the total proportion of time segregated averaged over twelve 4-minute simulations at points on a 21 × 21 grid with *PR* ∈ [5, 20] and Δ*f* ∈ [1, 22]. The proportion of time integrated *U*_int_ is defined by

$$U_{\text{int}}=\frac{V^{\text{int}}}{V^{\text{seg}}+V^{\text{int}}},$$ (2)

where *V* ^int^ is the total time that integrated is dominant (similarly *V* ^seg^ for segregated) across the twelve simulations at given (*PR*, Δ*f*)-values.

Fig 6B shows a grayscale map of *U*_int_ across the (*PR*, Δ*f*)-plane. We specify the integrated region by *U*_int_ > 0.95, the ambiguous region by 0.05 < *U*_int_ < 0.95 and the segregated region by *U*_int_ < 0.05. The 0.05 and 0.95 contours are plotted in Fig 6B. The model produces the correct organization with a predominance of integrated for small Δ*f*, segregated for large Δ*f* and ambiguous at intermediate ranges. Furthermore, the ambiguous region’s Δ*f*-range contracts at larger presentation rates, consistent with [2].

### Parametric dependence of perceptual dominance: model

**Fig 7:**
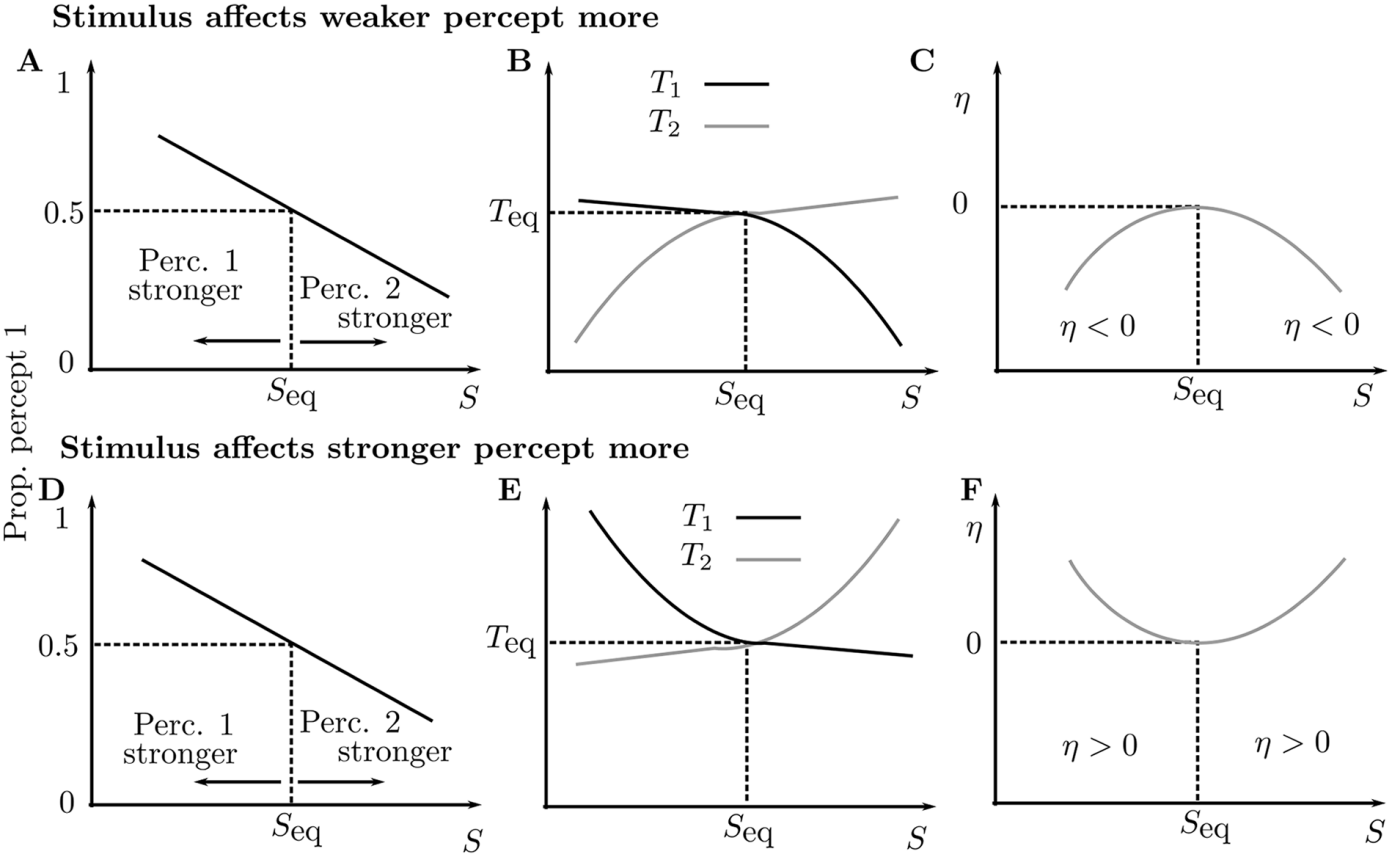
Scenarios for parametric dependence of perceptual dominance. Schematic diagrams illustrate how the mean percept durations may change as a stimulus parameter S is varied and dominance shifts gradually from percept 1 to percept 2. The upper row illustrates the weaker percept being affected more and the lower row the stronger percept being affected more. **A,D**: Proportion of time when percept 1 is dominant decreases monotonically through equidominance (0.5) (dashed lines) in both scenarios. When *S* < *S*_eq_ percept 1 is stronger, when *S* > *S*_eq_ percept 2 is stronger. **B,E**: Percept durations are equal (*T*_1_ = *T*_2_ = *T*_eq_) at equidominance (*S* = *S*_eq_) in both scenarios. When the weaker percept is affected more the lower branches decrease more on either side of equidominance (**B**). When the stronger percept is affected more the upper branches increase more on either side of equidominance (**E**). **C,F**: The measure *η* (defined by Eq (3)) is zero at equidominance (*S* = *S*_eq_) for both scenarios. It decreases on either side of equidominance when the weaker percept is affected more (**C**) and increases on either side of equidominance when the stronger percept is affected more (**F**).

Before presenting the model’s predicted behavior for the dependence of percept durations varying a stimulus parameter Δ*f*, we consider two different scenarios for a general experiment on bistable perception. The mean durations of two competing percepts are *T*_1_ and *T*_2_. Suppose that *S* is a parameter affecting the proportion of time *P*_1_ that percept 1 is dominant, and that *P*_1_ is close to 1 when *S* is small and decreases monotonically through 0.5 as *S* is increased. In Fig 7 panels A and D show this general relationship between *P*_1_ and *S*. When *S* = *S*_eq_ the percepts are dominant for an equal proportion of time (*P*_1_ = 0.5). We call *S*_eq_ the *equidominance point*, where *T*_1_ = *T*_2_. Panels B and E illustrate two different ways that *T*_1_ and *T*_2_ can vary to obtain this general relationship for *P*_1_. In the upper panels, the stimulus affects the weaker percept more, that is, either side of *S*_eq_ the percept with shorter duration (the *weaker percept*) changes more with *S*. For decreasing *S* < *S*_eq_, *T*_2_ decreases more than *T*_1_ increases and for increasing *S* > *S*_eq_, *T*_1_ decreases more than *T*_2_ increases. In the lower panels, the stimulus affects the stronger percept more, that is, for decreasing *S* < *S*_eq_, *T*_1_ increases more than *T*_2_ decreases and for increasing *S* > *S*_eq_, *T*_2_ increases more than *T*_1_ decreases.

We further distinguish between these three cases by defining *η* as the normalized total duration above equidominance

$$\eta \left(S\right)=\frac{T_{1}\left(S\right)+T_{2}\left(S\right)-2T_{\text{eq}}}{T_{\text{eq}}}.$$ (3)

This measure is negative when the weaker percept duration is affected more and positive when the stronger percept duration is affected more; see Fig 7C and 7F. Equivalently, one could show that the overall rate of alternation 1/*η* is minimal at equidominance when the weaker is affected more or maximal at equidominance when the stronger is affected more. The latter case was shown to be consistent with three visual bistable paradigms in [38].

**Fig 8:**
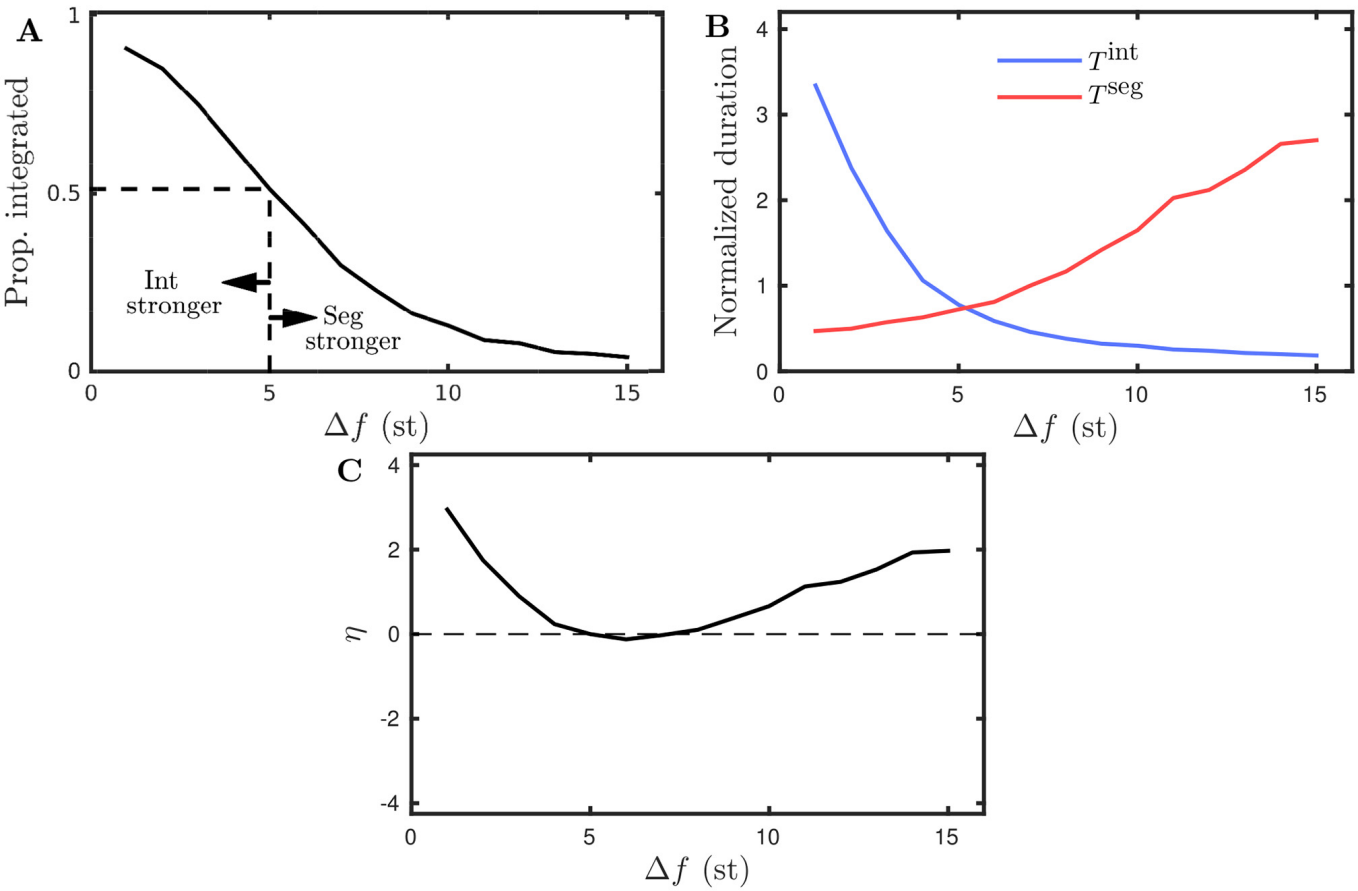
Parametric dependence of perceptual dominance: model prediction with (*e*_fix_, *i*_lcl_). **A**: Proportion integrated computed across 50 simulations of 240 s at each of 15 values of Δ*f* ∈ [1, 15] with fixed *PR* = 8 (this parameter range corresponds to the dashed vertical black line in Fig 6A). There is a shift from integrated being stronger to segregated being stronger with equidominance (indicated by dashed lines) at Δ*f* ≈ 5. **B**: Normalized durations integrated and durations segregated with a crossover at Δ*f* ≈ 5 where *T* ^int^ = *T* ^seg^. **C**: The measure *η* given by Eq (3), which equals 0 at equidominance. The results are consistent with Δ*f* affecting the stronger percept more (Fig 7D–7F).

We now make a comparison between the model and the scenarios proposed above. In particular, we are interested to see if the model predicts behavior that is consistent with gLPII, where the stronger percept is affected more than the weaker either side of equidominance. We recall that the model was set up and constrained to match pilot data for the distribution of percept durations and the van Noorden organization, as discussed in the preceding sections. The model’s ability to account for the available data provides confidence in its predictive power. Here we vary Δ*f*, a parameter that affects the proportion of time integrated in a way consistent with the examples from Fig 7, see Fig 8A. Fig 8B shows the normalized mean durations as a function of Δ*f*, where the durations for integrated are longer at small Δ*f* and the durations for segregated are longer for large Δ*f*, with equidominance reached at Δ*f* = 5. The normalization of durations, analogous to that used for the experimental data presented later, is explained in *Computation of normalized durations*. On each side of equidominance the upper branches increase more than the lower branches decrease, which is consistent with Fig 7E and Δ*f* affecting the stronger percept more than the weaker. This is further illustrated in Fig 7F, where *η* is positive either side of equidominance. We also note an asymmetry, with a sharper increase of the integrated durations for Δ*f* < 5 than in the segregated durations for Δ*f* > 5. The predicted behavior from the model is consistent with gLPII [38].

### Parametric dependence of perceptual dominance: experiment

**Fig 9:**
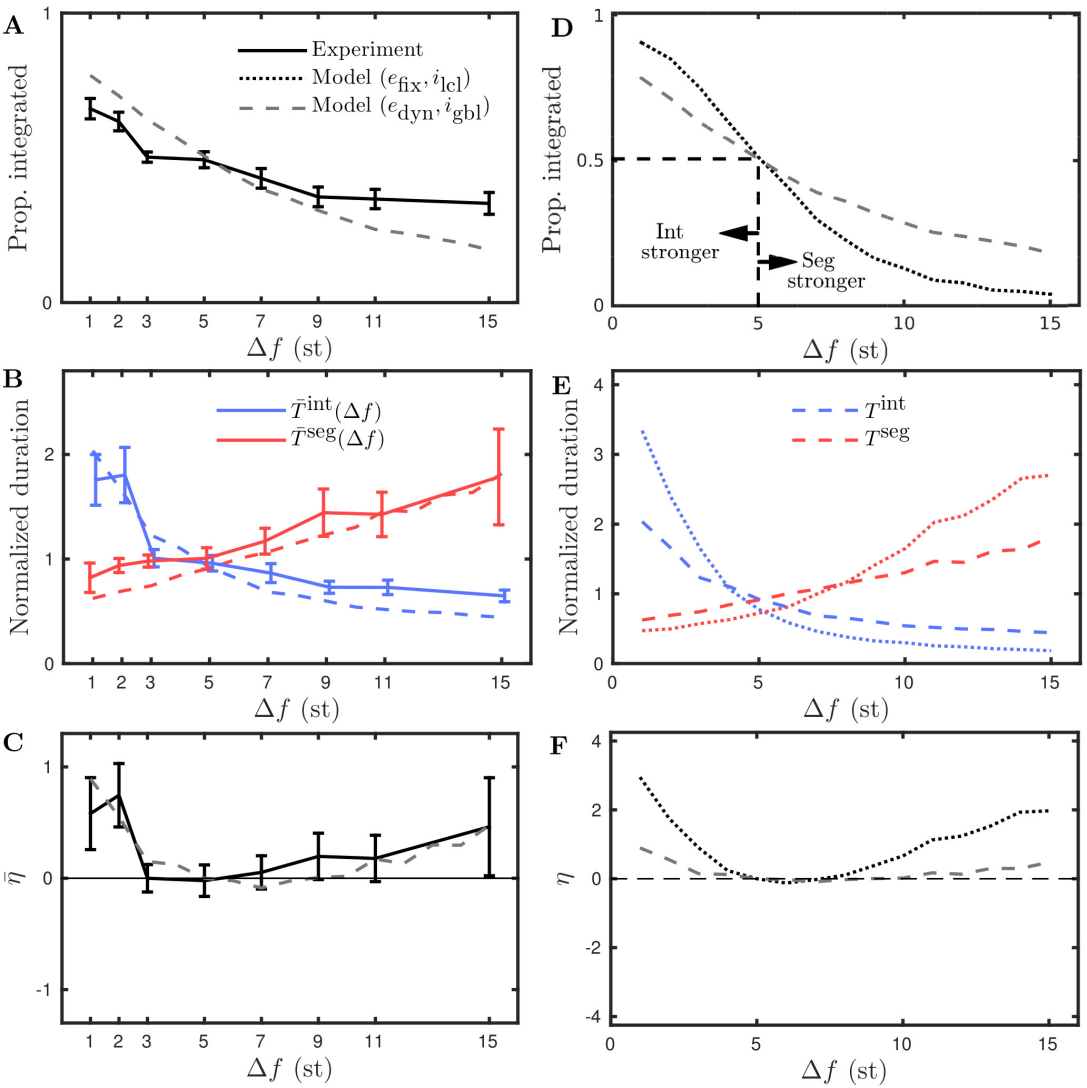
Parametric dependence of perceptual dominance: experiment. Comparison of experimental and computational results. **A–C**: Proportion integrated, durations integrated and segregated, and the measure *η* plotted against Δ*f*. Experimental data are solid curves with data points at the Δ*f*-values indicated on the x-axis, error bars show standard error of the mean with *N* = 15 subjects except at Δ*f* = 1 (*N* = 13) and Δ*f* = 15 (*N* = 14). Model data with dynamic recurrent excitation and global inhibition (*e*_dyn_, *i*_gbl_) plotted for comparison. **D–F**: Model data with (*e*_dyn_, *i*_gbl_) plotted with model data for fixed recurrent excitation and spatially localized inhibition (*e*_fix_, *i*_lcl_) for comparison.

We carried out behavioral experiments to characterize the effect of varying Δ*f* on the balance between the durations of the integrated and segregated percepts, allowing for a comparison with the model prediction and gLPII. As explained below we consider the durations of percepts following the first switch and the durations are normalized for each subject. In Fig 9A–9C solid curves with error bars show the three measures described and illustrated in Fig 7 for the auditory streaming experiment over the eight Δ*f*-values indicated on the x-axis. Fig 9A shows that for increasing Δ*f* the proportion of time integrated decreases monotonically; a one-way repeated measure ANOVA shows a significant effect (*F*(7, 77) = 24.656, *P* < 0.001, Greenhouse-Geisser corrected *P*-values are reported where appropriate, see *Repeated measures ANOVAs* for further details). Our data show that Δ*f* shifts the balance from integrated at low Δ*f* to segregated at large Δ*f*. Therefore, this experiment varying Δ*f* is suitable for a comparison with our qualitative modeling predictions and gLPII. Fig 9B shows that with increasing Δ*f* mean duration integrated ${\bar{T}}^{\text{int}}$ decreases and mean duration segregated ${\bar{T}}^{\text{seg}}$ increases with a crossover occurring between Δ*f* = 3 and Δ*f* = 5; a two-way repeated measure ANOVA shows a significant interaction between percept type and Δ*f* (*F*(7, 77) = 16.225, *P* < 0.001). In the analysis that follows we take the case Δ*f* = 5 as the equidominance point for the data, where integrated and segregated have approximately equal durations. Visual inspection indicates that either side of Δ*f* = 5 the upper branches of this X-shaped diagram increase more than the lower branches decrease. The measure *η* defined in Eq (3) tests this qualitative description. Fig 9C shows that *η* is positive either side of Δ*f* = 5 and shows that Δ*f* affects the stronger percept (percept dominant for larger proportion of time) more than the weaker either side of equidominance, compare Fig 9A–9C with Fig 7D–7F. A one-way repeated measures ANOVA on *η* with Δ*f* as factor does not reach significance (*F*(7, 77) = 0.878, *P* = 0.463). Further details and ANOVA tables reported in *Repeated measures ANOVAs*.

We found a substantial variation across individuals in their mean durations. For each subject a global mean duration *T* ^glob^ was computed across all trials and conditions (for summary statistics of *T* ^glob^, see *First durations and normalization*). One clear outlier, with *T* ^glob^ = 51.4 s (consistently longer than the group across all conditions) is excluded from further analysis. The remaining 15 subject’s *T* ^glob^-values span the range 2.6–14.8 s, which shows that some subjects switch much more rapidly than others. In the analysis that follows each subject’s durations are normalized by their *T* ^glob^. Furthermore, to avoid biasing the results towards the faster switchers who record more durations, each subject contributes a single mean integrated ${\bar{T}}^{\text{int}}\left(\Delta f\right)$ and mean segregated ${\bar{T}}^{\text{seg}}\left(\Delta f\right)$ score at each value of Δ*f*. The mean for each subject at each Δ*f* is taken across all durations from three repetitions. Refer to *Computation of normalized durations* for further details. We note that, before normalization the mean durations integrated and segregated at Δ*f* = 5 are respectively 7.86 s and 7.65 s, which is within the range reported in [19] where similar stimuli and procedure were used.

Existing studies of bistable (or multistable) auditory perception with long presentations (on the order of minutes) have established that first durations are typically longer than subsequent durations and should be treated separately [19, 21, 24]. In line with existing studies, across the range of Δ*f* values tested, we found the first durations to be consistently longer than subsequent durations, with the difference being largest for small Δ*f* and decreasing with Δ*f*, for further details see *First durations and normalization*. In the analysis presented, we study only the subsequent percept durations.

### Refined model better captures experimental results

The experimental data plotted in Fig 9A–9C agrees with the following qualitative predictions from the model that were illustrated in Fig 8A–8C (replotted in Fig 9D–9F as dotted curves, (*e*_fix_, *i*_lcl_) in the figure legend). Proportion integrated decreases monotonically with Δ*f*. Normalized durations integrated ${\bar{T}}^{\text{int}}$ and segregated ${\bar{T}}^{\text{seg}}$ form an X diagram with crossover at equidominance Δ*f* = 5 with the stronger percept (upper branches) increasing more than the weaker percept (lower branches) decrease either side of Δ*f* = 5. Accordingly the measure *η* is zero at Δ*f* = 5 and increases either side of Δ*f* = 5.

There are quantitative differences between the experiment and the model predictions from Fig 8A–8C. The model predicted a larger range of variation of proportion integrated than the experiment. The model predicted larger increases in duration for the stronger percept either side of equidominance and also larger decreases in the duration of the weaker percept either side of equidominance. Overall this results in the model predicting a stronger effect on *η* than was observed in the experiments.

Two mechanistic changes were made to the model that dramatically improve the quantitative fit between model and experiments, but do not alter the basic qualitative predictions. The first mechanistic change is a shift from localized inhibition that decays with Δ*f* to global inhibition independent of Δ*f* (compare *i*_lcl_ and *i*_gbl_ curves in Fig 2C). This change along with a reduction in input amplitude *I*_*p*_ from 0.525 to 0.425 improved the agreement between the upper branches in Fig 9B. The second mechanistic change introduces a minimum timescale for perceptual dominance immediately following a switch, which improves the agreement between the lower branches in Fig 9B. This is achieved by an increase in the strength of recurrent excitation whilst also introducing a slow synaptic depression. For further details of this extension to the model, see *Model equations and details*. We refer to this updated version of the model with dynamic recurrent excitation and global inhibition as (*e*_dyn_, *i*_gbl_). A direct comparison is made with the experimental data in Fig 9A–9C and with the earlier version of the model (*e*_fix_, *i*_lcl_) in Fig 9D–9F.

We note that the attained level of agreement between the model and experiment is not contingent on the normalization of percept durations. The normalization served to remove variability within subjects. To better see the effect of the normalization compare Fig 9B with non-normalized durations as given in *Computation of normalized durations*.

### Further predictions for constrained model

**Fig 10:**
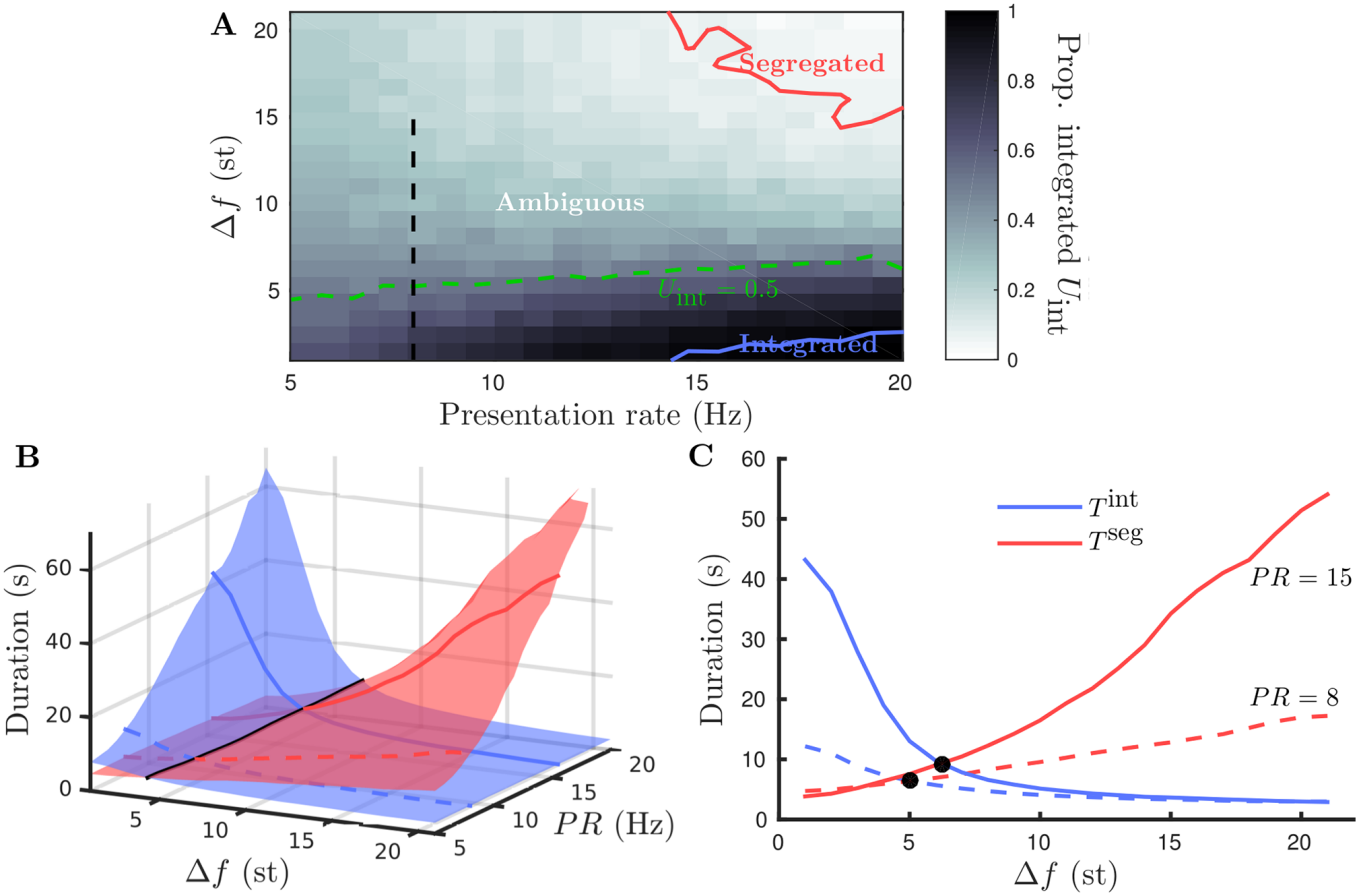
Perceptual organization for stimulus parameters in model (*e*_dyn_, *i*_gbl_). **A**: Proportion integrated *U*_int_ varying Δ*f* and *PR* plotted as a grayscale map. Solid are contours at *U*_int_ = 0.05 (red) and *U*_int_ = 0.95 (blue) demarcating regions where segregated and integrated are considered dominant, respectively. A dashed green contour in the ambiguous region indicates equidominance *U*_int_ = 0.5. Vertical dashed line at *PR* = 8 corresponds to the frequency difference sweep used in Figs 8 and 9. **B**: Surface plots of mean duration integrated and mean duration segregated (not normalized) across the same range as A. Black curve is the intersection of the two surfaces (equidominance). Dashed and solid curves indicate the cross sections plotted in C at *PR* = 5 and *PR* = 15, respectively. **C**: Durations integrated and segregated varying Δ*f* for fixed *PR* as indicated; in each case the equidominance point is a black dot.

After introducing modifications that allow the model to account for our experimental data, we can reevaluate the perceptual dominance assumption based on the van Noorden diagram. For the model with (*e*_dyn_, *i*_gbl_), Fig 10A shows a grayscale map of proportion integrated over a range of *PR* and Δ*f*-values. Solid curves show 95% boundary (bottom right corner), outside which integrated is considered as dominant and the 5% boundary (top right corner), outside which integrated is considered as dominant. In the model with (*e*_dyn_, *i*_gbl_), the qualitative organization is the same as for the model with (*e*_fix_, *i*_lcl_), but the regions of perceptual dominance are much smaller; compare Fig 10A with Fig 6B. The model with (*e*_dyn_, *i*_gbl_) (i.e. constrained by our experimental data) produces alternations over a much broader range of Δ*f* and *PR* than could be expected with the van Noorden perceptual dominance assumption.

The model predicts a stronger effect of gLPII at higher presentation rates. Fig 10B and 10C show durations integrated and segregated plotted over a range of Δ*f*- and *PR*-values. This model data has not been normalized, which will allow for future quantitative comparison with the predicted behavior. Dashed curves correspond to *PR* = 8 Hz, as plotted previously in Fig 9, and solid curves at larger *PR* = 15 Hz. The results show a modest increase in durations at equidominance and a slight upward shift in Δ*f* of the equidominance point with increasing *PR*. The weaker percept durations (see lower branches in panel C) are relatively unaffected by an increase in *PR*. The bias towards affecting the stronger percept durations (see upper branches in panel C) increases further at larger *PR*-values.

The most relevant comparison with published experiments could be with [24]. Results separating first and subsequent post build up durations showed a dramatic increase in mean duration (combined across percept types) for large Δ*f* and *PR*, and our model predicts similar behavior [24]. Values of Δ*f* below 4 st were not tested in [24] to see if a similar trend predict persists for large *PR* and small Δ*f*. Where integrated and segregated durations are reported separately in [24], no distinction is made between first and subsequent durations, precluding a direct comparison. Another study [21] with minutes-long presentations and a wide ranges of Δ*f* and *PR* tested did not distinguish between percept types, thus also precluding a direct comparison.

## Discussion

We developed and presented a neuromechanistic model of bistable auditory perception for the streaming paradigm of ABA- triplet stimuli. Our firing rate model accounts for the dynamics of alternations after the build up of streaming, matching the statistics from our psyshoacoustic experiments. The model’s architecture incorporates the tonotopic organization of auditory cortex. We suppose that the neuronal computation and our model’s units reside downstream from primary auditory cortex (A1); inputs mimic the tonotopic spread of activity in A1 and incorporate the transient (onset prominent) firing patterns known from physiological experiments [26, 28]. Alternations arise through the interplay of mutual inhibition, noise, and slow adaptation. This combination captures the characteristics of exclusivity, randomness, and inevitability as described by [35] and demonstrated for auditory bistability in [19]. Our model has some novel features: It incorporates NMDA-like recurrent excitation that supports some neuronal memory, enabling carry-over tone-to-tone within a triplet and across the tone gaps between triplets; percepts and dominance emerge naturally from the tonotopic architecture rather than having percepts pre-assigned to units that compete for dominance as in many previous models for perceptual bistability [33, 34]. With modeling and experiments we have confirmed that the auditory streaming paradigm conforms to a generalization of Levelt’s proposition II (gLPII): with tone frequency difference, Δ*f*, as a control parameter we find that on either side of equidominance the stronger percept durations are affected more than the weaker percept durations, in agreement with related experiments in bistable visual perception [38].

### Model of neuronal competition beyond A1

The neuronal circuits for competition and perceptual encoding are assumed to be located in downstream and to receive inputs from A1. Feature dependent activity and temporal forward masking can account for several aspects of auditory streaming build up as demonstrated in recordings from pre-attentive areas across a range of species, for example, studies of primate A1 [26–28], European starling forebrain [29] and guinea pig cochlear nucleus [30]. Spiking patterns in the European starling forebrain (assumed homologous to A1) have been shown to reflect stimulus characteristics but not perceptual decision [31]. Another study in ferrets has also demonstrated a disconnect between perceptual behavior in humans and the encoding of stimulus features in ferret A1 [43]. Recent fMRI studies (human subjects) provide evidence that links activation in both A1 and sub-cortical areas to perceptual reversals for streaming experiments [22, 44], however, the associated low temporal resolution cannot preclude this activation being a result of top-down modulation from higher auditory centers or other mixed/non-auditory areas. Behavioral and MEG studies (human subjects) with long stimulus presentations of streaming stimuli [18] and an informational masking paradigm [45] provide evidence that the auditory core encodes stimuli features independent of perceptual awareness, whilst perception is represented in longer latency responses associated with later stages of processing in non-primary auditory cortex [45]. Studies with human fMRI have shown that tonotopic organization extends beyond core regions (including A1) to some secondary auditory areas, see [46, 47] and [48] for a review. In our study we explored two possibilities, that the lateral inhibition in our competition stage depends on Δ*f* (i.e. tonotopic distance between the units), or that the inhibition is global and independent of Δ*f* (consistent with there being no tonotopy in the competition stage’s recurrent connections). Based on the available literature we are not able to pinpoint a more specific location for our model. We have proposed a minimal description of the competition involved in auditory bistability, which likely involves multiple brain areas beyond A1.

### Model formulation and fitting of parameters

The reduction of a continuous representation of a feature space to individual units encoding different percepts was first proposed in [32] and simplified models where the competing percepts are assumed have become relatively standard in modeling studies of perceptual bistability (see, for example, [33, 34, 42, 49]). In these models inputs are percept specific rather than incorporating a realistic feature dependence. In our model, the competing percepts arise from the tonotopic stimulus feature dependence without assuming that abstracted populations encode each percept. This, along with the periodic nature of the inputs distinguishes our work from typical competition models. Most existing computational models of auditory streaming focused on the build up phase and, although some include a hybrid of auditory pathway physiological detail and electrical signal processing [14, 17], they are typically abstract in construction [11, 12, 15, 16]. Both build up and subsequent alternations were modelled in [24] with competition taking place between abstracted units representing patterns formed in the build up phase. The focus on alternations after the build up phase, the neuromechanistic formulation that we propose and the emergence of competing percepts within the tonotopic organization distinguish our work from existing auditory models.

Model parameters controlling the balance between adaptation and noise were tuned to match the statistics of perceptual durations in terms of mean durations, fit to a log-normal distribution and coefficient of variation. The level of agreement obtained demonstrates that the dynamics of auditory bistability were accurately captured by the model. Model parameters controlling the spread of input and range of inhibitory connections in tonotopic space were constrained based on a starting hypothesis that van Noorden’s proposed regions of perceptual dominance [2] are also applicable to the perceptual bistability we study here. In order to obtain the van Noorden organization a tonotopic dependence of lateral inhibition within our model’s competition stage was included. The assumption of a tonotopic dependence, beyond that inherited from A1 through its inputs, in the lateral recurrent connections in the competition stage is linked to the hypothesis for the van Noorden organization. Below we discuss this assumption in light of our own data whilst highlighting a number of differences between van Noorden’s experimental paradigm and the situation considered here. Nevertheless, the reproduction of the correct perceptual organization suggests that stimulus features have been accurately captured along with the dynamics of alternations. On this basis the model was used to predict the relationship between frequency difference Δ*f* and the relative durations for the integrated and segregated percepts. Data from experiments designed to test the model prediction were later used as a more concrete means to constrain the model.

### Experiment to test Levelt’s II in auditory bistability

We asked whether gLPII extends to auditory bistability. We chose Δ*f* as a control parameter since it affects the proportion of dominance between the percepts [21, 24]. Indeed, our model satisfies gLPII (compare Fig 8 with schematics in Fig 7D–7F), which cannot be generically expected given differences in mechanisms and architecture with the models that show gLPII-like behavior for visual bistability [32, 38, 50]. Satisfaction of gLPII for auditory bistability was a joint prediction from our model and the literature on bistable perception.

Results from our psychophysical experiments also satisfied gLPII (Fig 9A–9C), supporting the predictive power of our basic model. Repeated measures ANOVAs demonstrated a significant effect of varying Δ*f* on proportion of time integrated, a significant interaction between percept type and Δ*f*, but no significant effect of varying Δ*f* on *η*. Given that *η* was positive away from equidominance, we can conclude that auditory bistability conforms to gLPII, however, the bias towards affecting the stronger percept is modest. A recent study [24] reported durations of integrated and segregated percepts over a range of Δ*f*-values, but not a sufficient number of conditions were tested at a fixed presentation rate to allow for a direct comparison with gLPII.

In order to improve the quantitative agreement between the model and experiment, we made two mechanistic changes and minor parameter adjustments in the model. Firstly, by relaxing the hypothesis of conforming to the qualitative van Noorden organization and shifting to global inhibition the overestimation was reduced for durations of the stronger percept, see Fig 9E. The implication is that tonotopic dependence of inhibition is not required at the level where percepts are encoded although localization of inhibition and excitation are present in A1 for conveying stimulus features and dependence on Δ*f*. We searched many different parameter combinations testing the relative balance between the Δ*f*-footprints of inhibition and input but did not identify another means, within the scope of the model’s features, to account for the experimental data. In the conception of our model, we assumed that the competition in our model takes place at a stage with tonotopy-dependent inhibition between units (plausible for non-primary auditory areas). However, given that our model better accounts for our experimental findings with (tonotopy independent) global inhibition, it is also plausible that the competition take place in a non-tonotopically organised area of auditory cortex or a non-auditory area downstream of A1 (for example, intraparietal sulcus (IPS) as implicated in an fMRI study of auditory streaming [51]). Although tonotopy is still inherited through the inputs to our competition stage, it does not necessarily play a role in the recurrent synaptic inhibition. Secondly, we increased the strength of recurrent excitation whilst introducing a slow synaptic depression that could prolong a dominance duration beyond a minimum value. The modification of the recurrent excitation mechanism effectively prevents immediate switches away for a unit that has recently become active. This change reduced the underestimation of durations for the weaker percept, see Fig 9E. Self excitation also played a role in reducing the probability of short durations in [24].

### van Noorden organization

Regions of temporal coherence (dominance of integrated), fission (dominance of segregated) and ambiguity over ranges of Δ*f* and *PR* were established in [2] and the resulting van Noorden diagram has become a key point of comparison in much of the research that has followed. However, comparisons to the van Noorden diagram are often made in experiments and models where several features particular to van Noorden’s experiments are not take into account. The procedure used by van Noorden included a slowly increasing or decreasing frequency difference, and subjects were instructed to attend to (“hold”) a specific interpretation during presentation. Trials were terminated after the first perceptual switch. Although the van Noorden diagram has provided a rough, qualitative overview of perceptual organization, a full comparison with these experiments will await the development and application of a model that can treat early and later phases of streaming, time varying stimulus parameters and that includes some mechanism for attention.

We used the organization of perceptual dominance and ambiguity as described/established by van Noorden [2] as a qualitative guide in the initial determination of our model’s parameter values, particularly those that control tonotopic spread of input (from A1) and the tonotopic footprint for the strength of inhibition. The data from our subsequent psychoacoustics experiments (to investigate gLPII) provided a more concrete means to constrain the model. Once constrained by our experimental data, the model predicted that alternations occur over a much broader range of Δ*f* and *PR* than expected from the original van Noorden diagram. The hypothesis that the post build-up phase of streaming conforms to the van Noorden organization was not satisfied in a qualitative way. Our results support recent experiments that found alternations taking place over the entire range of Δ*f* and *PR* typically tested in streaming experiments [21]. A further testable model prediction, which should be the subject of future experiments, is that the bias towards affecting the stronger percept durations either side of equidominance increases with presentation rate.

### Attention

Could attention be thought of as a top-down modulation of the effective input to neuronal groups whose dominance corresponds to the attended percept? Attention can significantly affect the proportion of dominance during alternations between the competing percepts [19]. Interestingly, although the proportion increased for the attended percept the effect was achieved by decreased mean durations of the weaker (unattended) percept; results were reported only for an equidominance condition (Δ*f* = 5 st). If there is a parallel it would be with the hypothetical case of Fig 7A–7C, where the weaker percept is affected more than the stronger percept. In contrast, our study has shown that stimulus manipulations affected the stronger percept more than the weaker percept on either side of equidominance (Fig 9)—consistent with gLPII (Fig 7D–7F). Our observations suggest an opposite effect in auditory bistability for attention and Δ*f*-manipulations. We conclude that in order to model effects of attention we should consider more than static manipulations of stimulus parameters. Simply increasing the stimulus strength to favor the attended percept will be inadequate. Rather, we suggest that inputs should be dynamic and depend on perceptual state. An exploration of the neural mechanisms underlying attention effects in auditory streaming will be the focus of future work.

### Toward a more general modeling framework

In most studies with the ABA- paradigm, subjects report integrated and segregated percepts, but some psychoacoustic studies have documented other interpretations [21, 52–54]. Our model’s dynamic responses were categorized with a simple criterion to be either integrated or segregated. The categorization could be extended to include the possibility of the A or B tones being in the foreground within the segregated percept as was considered in recent psychoacoustic experiments [53]. The extension of phenomenological, percept-based, competition models to tristable perception was considered in [49] for visual motion plaids, and a similar extension for auditory stimuli would be of interest. We did not formulate a neuromechanistic description for distinguishing a wider variety of patterns (as reported in experimentally [54]), which remains an outstanding challenge for modeling auditory perception (see [24] for a proposed algorithmic approach).

The temporal coherence in the relative onsets of the A and B tones in the streaming paradigm can have a significant effect on perception [55]. As has been demonstrated for short stimulus presentations, there is a bias towards grouping of stimulus elements with a common onset even when they separated by large frequency differences [43] (see [56] for an example with harmonicity-based ambiguity where this is not the case). We think our framework could be extended to consider coherence by including another feature dimension for, say, assessing temporal coincidence.

Recent imaging studies have shown changes in activation around the time of auditory perceptual switches not only in auditory areas, but in non-auditory areas such as IPS [51] and cerebellum [23], and in subcortical regions such as the medial geniculate body [23] and inferior colliculus [44]. Although it is likely that subcortical areas play an important role in early stimulus adaptation and build up, their exact role in auditory bistability is still far from clear. The extension of our model to incorporate a hierarchical structure with descending feedback and further subcortical detail would allow for an exploration of whether activity in subcortical areas is a driving force in perceptual alternations or the result of modulation through descending connections.

The present model has been specifically designed around a stimulus involving two sets of tones at a fixed frequency separation. It is formulated with discrete neuronal units that are tuned to tone frequencies A, B or the intermediate (A + B)/2. The amount of input going to each unit and the inhibition between the units was defined through continuous functions. This description could be directly extended to a continuous representation of tonotopic space that would allow for an arbitrary number of inputs and for inputs with Δ*f* varying over time. Such a representation was used in the modelling study [16] to look at build up and at rising/falling tone sequences perceived as *crossing* or *bouncing* as reported in experiments [2]; elsewhere, the choice of a continuous feature has proved effective in capturing the dynamics of multistable motion perception [57]. A model with a continuous tonotopic representation would provide a basis to look more closely at the experiments of van Noorden involving Δ*f*-time-varying stimuli and bridge the gap with the fixed stimuli considered here.

### Conclusion

Our study’s foremost contribution is a physiologically-based model of auditory bistability that captures key features of the perceptual alternation dynamics for the ABA- streaming paradigm. Our formulation explicitly incorporates sound-evoked A1 responses as input to a competition network. Perceptual states are identified in the neuronal dynamics rather than pre-assigned to phenomenological units. The inclusion of NMDA-like synaptic dynamics enables temporal binding from one triplet to the next in order to deal with gaps in the periodic sound input. We applied the model to predict the dependence of mean dominance durations on the tone frequency difference Δ*f* and carried out psychoacoustic experiments for comparison. Based on our findings we refined the model to include slow depression of the NMDA excitation and a broadened footprint for inhibition. The quantitative agreement between the model and experiments across several important signatures, including the steady state statistics of percept durations, demonstrates the model’s success in capturing the dynamics of auditory bistability.

In parallel with the model development we established in our experiments that generalized Levelt’s Proposition II holds for the alternations in auditory bistability that follow the first perceptual switch. We showed that varying Δ*f* away from equidominance increases the duration of the stronger (dominant a larger fraction of time) percept rather than that of the weaker. These findings further establish a further commonality between auditory and visual bistable dynamics [38]. Although the measured effect in favor of increasing the stronger percept duration was small, it was opposite to that of attention as shown in [19] (strongly in favor of decreasing the weaker percept’s duration). Our model provides a platform to further investigate these contrasting effects and to explore the mechanisms of attention in auditory streaming.

## Materials and methods

### Ethics statement

The psychoacoustics experiments were approved by the University Committee on Activities Involving Human Subjects at New York University (IRB #12-8810).

### Model equations and details

We consider a three population network—a discrete idealization of a tonotopically organized array. Two populations pool inputs from regions of A1 centred at locations with best frequenices A and B, whilst a third population pools input centred at a tonotopically intermediate location, say (A + B)/2; see Fig 2.

The firing rate variables *r*_*k*_ are indexed by *k* = {*A*, *AB*, *B*} for each population shown in Fig 2A with the associated adaptation *a*_*k*_ and recurrent excitation *e*_*k*_ variables (note that, throughout the paper, the symbol “*e*” is used exclusively for excitation variables and associated constants whilst the symbol “exp()” is used for the exponential function). The system of first order differential equations is as follows:

$$\begin{array}{cc} \tau_{r}{\dot{r}}_{\text{AB}} & =-r_{\text{AB}}+F(\beta_{e}d_{\text{AB}}e_{\text{AB}}-C_{i}\left(0\right)r_{\text{AB}}-C_{i}\left(\Delta f/2\right)\left(r_{A}+r_{B}\right)\\ & -ga_{\text{AB}}+w\left(\Delta f/2\right)\left(I_{A}+I_{B}\right)+\chi_{\text{AB}}),\\ \tau_{r}{\dot{r}}_{A} & =-r_{A}+F(\beta_{e}d_{A}e_{A}-C_{i}\left(0\right)r_{A}-C_{i}\left(\Delta f/2\right)r_{\text{AB}}-C_{i}\left(\Delta f\right)r_{B}\\ & -ga_{A}+I_{A}+w\left(\Delta f\right)I_{B}+\chi_{A}),\\ \tau_{r}{\dot{r}}_{B} & =-r_{B}+F(\beta_{e}d_{B}e_{B}-C_{i}\left(0\right)r_{B}-C_{i}\left(\Delta f/2\right)r_{\text{AB}}-C_{i}\left(\Delta f\right)r_{A}\\ & -ga_{B}+I_{B}+w\left(\Delta f\right)I_{A}+\chi_{B}),\\ \tau_{a}{\dot{a}}_{\text{AB}} & =-a_{\text{AB}}+r_{\text{AB}},\\ \tau_{a}{\dot{a}}_{A} & =-a_{A}+r_{A},\\ \tau_{a}{\dot{a}}_{B} & =-a_{B}+r_{B},\\ \tau_{e}{\dot{e}}_{\text{AB}} & =-e_{\text{AB}}+r_{\text{AB}},\\ \tau_{e}{\dot{e}}_{A} & =-e_{A}+r_{A},\\ \tau_{e}{\dot{e}}_{B} & =-e_{B}+r_{B},\\ \tau_{d}{\dot{d}}_{\text{AB}} & =-d_{\text{AB}}+\left(1-\kappa \;r_{\text{AB}}\right),\\ \tau_{d}{\dot{d}}_{A} & =-d_{A}+\left(1-\kappa \;r_{A}\right),\\ \tau_{d}{\dot{d}}_{B} & =-d_{B}+\left(1-\kappa \;r_{B}\right), \end{array}$$ (4)

with time constants *τ*_*r*_ (cortical), *τ*_*a*_ (spike frequency adaptation), *τ*_e_ (NMDA-excitation) and *τ*_*d*_ (slow depression of excitation). Each population shares a common firing rate function *F* is given by

$$F\left(u\right)=\frac{1}{1+\exp\left(k_{F}\left(-u+\theta_{F}\right)\right)},$$ (5)

where *θ*_*F*_ is a threshold parameter and *k*_*F*_ is a slope parameter, see Fig 2D. The strength of adaptation is *g*.

Inhibition is assumed to be instantaneous (*τ*_*i*_ = 0 in $\tau_{i}{\dot{i}}_{k}=-i_{k}+r_{k}$ for each *k*) such that the inhibitory terms in Eq (4) appear as proportional to the *r*-variables (*i*_*k*_ = *r*_*k*_). Simulations were run (not shown) to verify that this simplification did not have a significant effects on the model’s dynamics. In the first phase of model simulations the strength of lateral inhibition is assumed to have Gaussian decay with Δ*f*

$$C_{i}\left(\Delta f\right)=\beta_{i}\exp(-\frac{\Delta f^{2}}{2\sigma_{i}^{2}}),$$ (6)

where the inhibition strength is *β*_*i*_ with decay constant *σ*_*i*_; the symbol *i*_lcl_ is used to refer to this case. In the second phase of the model simulations inhibition is assumed to be global such that *C*_*i*_(Δ*f*) = *β*_*i*_ (equivalently *σ*_*i*_ → ∞ in Eq (6)); the symbol *i*_gbl_ is used to refer to this case.

Recurrent excitation is assumed to occur on an intermediate timescale *τ*_e_ (representative of NMDA synapses) between *τ*_*r*_ and *τ*_*a*_. Excitation is assumed to be local only with strength *β*_*e*_ > *β*_*i*_ such that there is net local excitation for each unit. In the first phase of the modeling results the variables *d*_*k*_ are frozen at *d*_*k*_ = 1 (*κ* = 0) in (4); the symbol *e*_fix_ is used to refer to this case. In the second phase of the modeling results the strength of the recurrent excitation is increased from *β*_*e*_ = 0.7 to *β*_*e*_ = 0.85 and a slow (*τ*_*d*_ = 3 s) synaptic depression with strength *κ* = 0.25 is turned on; the symbol *e*_dyn_ is used to refer to this case. The effect of increased excitation strength coupled with a slow synaptic depression is to introduce a minimum active phase (i.e. a minimum percept duration) once a given *r*_*k*_ becomes dominant.

The spread of input is defined via the weighting function

$$w\left(\Delta f\right)=I_{p}\exp(\frac{-\Delta f}{\sigma_{p}})$$ (7)

where *σ*_*p*_ is a spatial decay parameter and *I*_*p*_ is the pulse amplitude. The input terms *I*_A_ and *I*_B_ appearing in Eq (4) are defined by Eq (8). The particular form of the periodic inputs are based on recorded responses from A1 with such ABA- stimuli [28]; see Fig 3. We capture the basic form of these responses with a pair of onset response functions, one with larger amplitude and early rise that captures the initial onset and a second with smaller amplitude and late rise that captures the plateau:

$$I\left(t\right)=H\left(t\right)\;[\frac{\exp(2)}{\alpha_{1}^{2}}t^{2}\exp(\frac{2t}{\alpha_{1}})+\Lambda_{2}\frac{\exp\left(2\right)}{\alpha_{2}^{2}}t^{2}\exp(\frac{2t}{\alpha_{2}})],$$ (8)

with plateau amplitude fraction Λ_2_ and rise times *α*_1_ < *α*_2_; see Fig 3 (thick black curve). The constant term $\frac{\exp\left(2\right)}{\alpha_{\left\{1,2\right\}}}$ normalises the amplitude at *t* = *α*_{1,2}_ of the individual onset functions to 1. A standard Heaviside function *H* ensures no response before an input tone at *t* = 0. Rise times of *α*_1_ = 15 ms and *α*_2_ = 82.5 ms and an amplitude Λ_2_ = 1/6 were chosen to approximately match the rise time and relative onset-to-plateau ratio observed in [28].

Additive noise is introduced with independent stochastic processes *χ*_A_, *χ*_B_ and *χ*_AB_ added to the inputs of each population as in [34] and [58]. Input noise is modeled as an Ornstien-Uhlenbeck process:

$$\dot{\chi_{k}}=-\frac{\chi_{k}}{\tau_{d}}+\gamma \;\sqrt{\frac{2}{\tau_{X}}}\xi_{k}\left(t\right),$$ (9)

where *τ*_X_ = 100 ms (a standard choice [34, 58]) is the timescale, *γ* the strength and *ξ*(*t*) a white noise process with zero mean. Note these terms appear inside the firing rate function *F* such that firing rates *r*_*k*_ remain positive and do not exceed 1.

All model parameters defined throughout this section are given in Table 1. The values of *θ*_*F*_ and *k*_*F*_ are standard for neuronal competition models [34, 58]. The input shape parameters Λ_2_, *α*_1_ and *α*_2_ are estimated from [28]. In the first modeling phase (*e*_fix_, *i*_lcl_), the Δ*f*-dependent functions controlling the spread of input (Eq (7)) and decay of lateral inhibition (Eq (6)) were empirically chosen to produce a van-Noorden like organization for the stimulus parameters Δ*f* and *PR* as shown in Fig 6. The parameters *I*_*p*_, *σ*_*p*_ and *σ*_*i*_ were chosen accordingly. In the second phase of the modeling (*e*_dyn_, *i*_gbl_), these were readjusted and a slow synaptic depression introduced (with parameters *κ* and *τ*_*d*_) to better account for our experimental data. The strength parameters for adaptation *g*, noise *γ*, inhibition *β*_*i*_ and excitation *β*_*e*_ were set to provide a balance between adaptation and noise [34], to produce the correct mean durations and switching statistics to match our pilot data, see Fig 5. The cortical timescale (*τ*_*r*_ = 10 ms) and the spike frequency adaptation timescale (*τ*_*a*_ = 1,400 ms) fall within typical ranges [59]; similar values have been used in competition models [34, 58]. The NMDA-like timescale (*τ*_e_ = 70 ms) for excitation is motivated from [60, 61] and similar values have been used in working memory and decision making models [62, 63]. In the second phase of the modeling results, excitation is assumed to decay through slow synaptic depression on a timescale *τ*_*d*_ = 3,000 ms, see below. Slow depression on the order several seconds has been reported in primary visual cortex [64] and several slow timescales for adaptation, also on the order of several seconds, have been reported in auditory cortex responses [65].

### Stimuli and procedure for psychoacoustic experiments

The stimuli consist of repeating 125 ms pure tone ABA- triplets where the ‘-’ indicates a silence also lasting 125 ms; each ABA- sequence is 0.5 s in duration (similarly, 125 ms tones were used in [28] and 120 ms tones were used in [19]). The higher frequency A tones are a variable Δ*f* semitones above the lower frequency B tones. Cosine squared ramps are used with 5 ms rise and fall times. During 4 minute trials the tone sequence is played binaurally through etymotic headphones at 65 dB SPL. Eight Δ*f* conditions are used Δ*f* ∈ {1, 2, 3, 5, 7, 9, 11, 15}, with each 8-trial block consisting of a single presentation of each condition. The frequencies used for the A and B tones are taken to be semitone intervals between 392 Hz and 932 Hz, a range spanning 15 semitones. Within a trial block a total of 16 frequencies are used, two for each Δ*f* condition, such that no frequency appears twice within the block; see Table 2. In this way, irrespective of the order of presentation of the 8 conditions, no specific frequency is repeated from one presentation to the next so as to avoid any residual effects of adaptation between trials. Furthermore, a minimum 30 s interval between trials (and 180 s after the fourth trial) was used after which subjects could the next trial when ready. The increments of Δ*f* have been chosen to give a good coverage of the range of responses based on trial data.

**Table 2:** Frequency conditions used.

Δ*f* (st)	A (Hz)	B (Hz)
1	622.3	587.3
2	640.5	570.6
3	659.3	554.4
5	698.5	523.3
7	740.0	493.9
9	784.0	466.2
11	830.6	440.0
15	932.3	392.0

Sixteen subjects (9 female) with a mean age 24 years took part in the experiment for modest reimbursement. Procedures were in compliance with guidelines for research with human subjects and approved by the University Committee on Activities Involving Human Subjects at New York University. All subjects provided written informed consent. Each subject completed three eight-trial blocks giving a total of 24 trials per subject. An 8 × 8 latin square design was used to determine the order of conditions for each subject and block repetition. Six randomized and unique grids were generated (two blocks of 8 subjects and three repetitions). The blocks were run across two sessions on different days, one block in the first session, two blocks in the second session.

Subjects sat in an acoustically shielded chamber and indicated their perceptual responses with button presses on a keyboard. In a 2AFC task subjects were instructed to report the integrated percept when they heard the A and the B tones together in an alternating or galloping rhythm and the segregated percept when they heard two separate streams, one with only A tones and one only B tones. The percepts were explained to the subjects with auditory and visual illustrations to ensure that the subjects understood the two interpretations and could clearly distinguish between them. Subjects were instructed to passively report their percepts without attempting to hear one perceptual organization over another. Subjects reported their percepts by holding specific keys associated with of the percepts. The state of the two response buttons was recorded with a sampling rate of 100 Hz.

In this paper we considered bistability between integrated and segregated percepts for ABA- triplets, using a 2AFC task in our experiments. In studies where response keys are provided for integrated and segregated and subjects are instructed to press neither key when their responses are “indeterminate” such responses are recorded for a very small fraction of presentation time [19, 24]. Here the task was 2AFC and no instruction for “indeterminate” responses was provided. We chose to use a simplified task given the wide range of Δ*f*-values tested. Furthermore, in order to make a direct comparison with gLPII, it was necessary to impose categorization between only two competing interpretations as done in the three visual paradigms considered in [38].

Durations shorter than 0.5 s (one triplet) were excluded from the analysis. Across all conditions neither key was depressed for 1.4% of total presentation time (including the time immediately after stimulus onset before any key is pressed) and both keys were depressed 0.9% of total presentation time. Times with neither or both keys depressed were at the transitions between the two percepts (any trials where neither or both keys were depressed for more than 10 s were inspected visually). Given the 2AFC task, each percept duration was computed from the button press onset associated with one percept type up to the button press onset of the opposite percept type. The final (incomplete) duration was discarded for each trial.

### First durations and normalization

**Fig 11:**
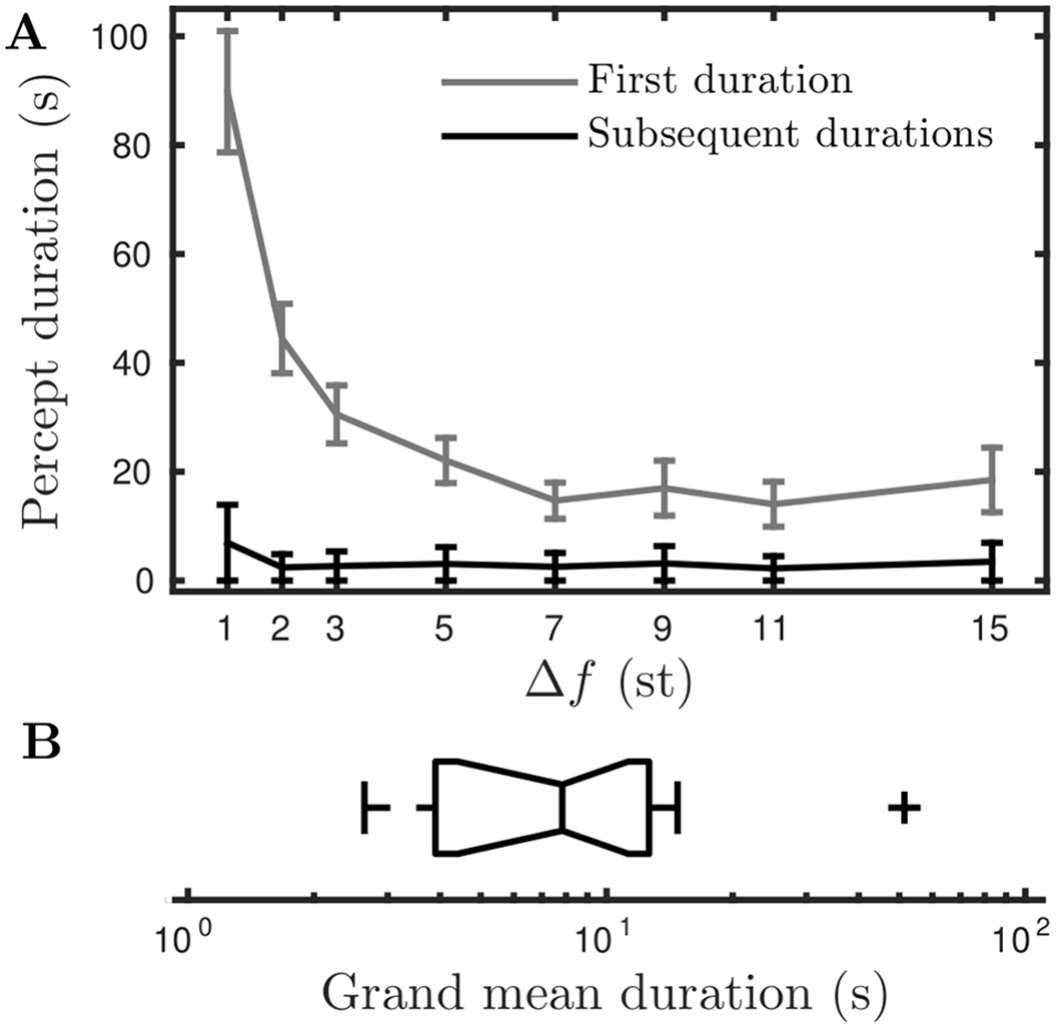
First and subsequent durations, summary statistics of the subject’s grand mean durations. **A**: Mean first percept duration and mean subsequent percept duration for all durations combined across both percept types, *N* = 16 subjects and *R* = 3 repetitions. Error bars show standard error of the mean. **B**: Standard Tukey box plot of *T* ^glob^ for *N* = 16 subjects (box shows quartiles, whiskers are most extreme data points within 1.5 × iqr of the upper and lower quartiles, “+” are individual outliers).

In an initial phase of our analysis, we establish that first durations are longer than subsequent durations across the range of Δ*f*-values tested. At each condition we look at, separated our into first and subsequent, the mean duration taken across both percept types (integrated of segregated), *N* = 16 subjects and 3 repetitions. For the first percept this gives 48 first durations at each condition and for subsequent percepts there is a variable number of durations at each condition (mean 224 individual durations). The results are plotted in Fig 11A where, consistent with the literature, first duration curve is well separated from the subsequent durations curve across all conditions, the different being largest for small Δ*f* and decreasing with Δ*f*. In all the analysis that follows the first duration is excluded.

We next look at how subjects responses are distributed in terms of their global mean percept duration *T* ^glob^, grouping data across all conditions, see (10). This information can be used to identify any outliers and will be used to normalize responses across subjects. For each of 16 subjects we take the mean percept duration taken across both percept types, all repetitions and all Δ*f* conditions. We plot summary statistics of the subject distribution of *T* ^glob^-values in Fig 11B. One clear outlier, with *T* ^glob^ = 51.4 s (consistently longer than the group across all conditions) is excluded from further analysis. The remaining 15 subject’s *T* ^glob^-values spans the range 2.6–14.8 s. The result is that many more durations are recorded from subjects with small *T* ^glob^ (fast switchers) than large *T* ^glob^ (slow switchers). In further analysis, if we were to combine data across subjects including all durations, any results would be skewed towards fast switchers who contribute more durations. To account for this, in the analysis that follows, each subject contributes a mean integrated and a mean segregated duration averaged across all durations for the three repetitions at each condition. Other measures such as proportion of time integrated are computed in a similar fashion. Furthermore, due to the large range of *T* ^glob^-values, representing a near-order-of-magnitude difference from smallest to largest, we also scale each subject’s durations by *T* ^glob^ before combining across subjects. Further details of the normalization are given in *Computation of normalized durations*.

### Computation of normalized durations

**Fig 12:**
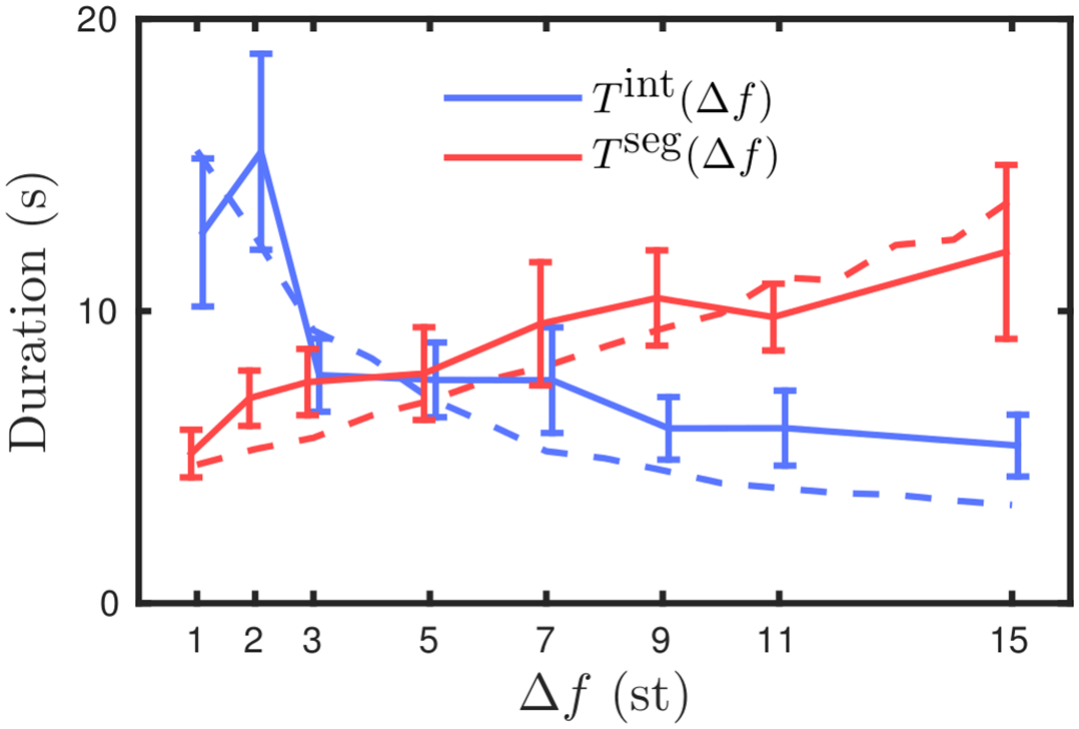
Non-normalized percept durations. Durations integrated and segregated without normalization plotted against Δ*f* for experiment and model with (*e*_dyn_, *i*_gbl_), as Fig 9B.

The normalization of percept durations, as described below, was used to remove some of the subject variability in experiments. For reference, the percept durations for the experiment and model with (*e*_dyn_, *i*_gbl_) are plotted without normalization in Fig 12. To see the effect of the normalization compare Fig 9B and 9C with Fig 12A and 12B.

For the experiment, we index *N* subjects by *n* = 1, …, *N*, *R* = 3 repetitions by *r* = 1, …, *R* and *C* = 8 conditions by *c* = 1, …, *C*. For each of the *N* × *R* × *C* trials the we index all *D* durations (integrated and segregated) recorded in that trial by *d* = 1…*D* or separately the *D*_int_ integrated durations by *d*_int_ = 1, …, *D*_int_ and the *D*_seg_ segregated durations by *d*_seg_ = 1, …, *D*_seg_ where *D* = *D*_int_ + *D*_seg_. The first duration and (incomplete) last duration are excluded. On a given trial all durations *T*_*n*, *r*, *c*, *d*_ can be indexed by *d* or indexed separated into durations integrated $T_{n,r,c,d_{\text{int}}}^{\text{int}}$ and durations segregated $T_{n,r,c,d_{\text{seg}}}^{\text{seg}}$.

For a given subject *k* the global mean percept duration is taken across *R* repetitions, *C* conditions and *D* durations

$$T_{k}^{\text{glob}}=\frac{1}{RCD}\sum_{r=1}^{R}\sum_{c=1}^{C}\sum_{d=1}^{D}(T_{k,r,c,d}).$$ (10)

Summary statistics of *T* ^glob^ are plotted in Fig 11B for *N* = 16 subjects.

For a given subject *k* and condition *j* the normalized mean duration integrated across R repetitions is

$${\bar{T}}_{k}^{\text{int}}\left(j\right)=\frac{1}{RD_{\text{int}}}\sum_{r=1}^{R}\sum_{d_{\text{int}}=1}^{D_{\text{int}}}(\frac{T_{k,r,j,d_{\text{int}}}^{\text{int}}}{T_{k}^{\text{glob}}}),$$ (11)

and, similarly, the noramlized mean duration segregated is

$${\bar{T}}_{k}^{\text{seg}}\left(j\right)=\frac{1}{RD_{\text{seg}}}\sum_{r=1}^{R}\sum_{d_{\text{seg}}=1}^{D_{\text{seg}}}(\frac{T_{k,r,j,d_{\text{seg}}}^{\text{seg}}}{T_{k}^{\text{glob}}}).$$ (12)

For a given condition *j* the the normalized mean duration integrated across subjects is

$${\bar{T}}^{\text{int}}\left(j\right)=\frac{1}{N}\sum_{k=1}^{N}{\bar{T}}_{k}^{\text{int}}\left(j\right),$$ (13)

and the standard error of the mean is the standard deviation of ${\bar{T}}_{k}^{\text{int}}\left(j\right)$ divided by $\sqrt{N}$. Similarly,

$${\bar{T}}^{\text{seg}}\left(j\right)=\frac{1}{N}\sum_{k=1}^{N}{\bar{T}}_{k}^{\text{seg}}\left(j\right).$$ (14)

An analogous normalization was used for the model data plotted across a range of Δ*f*-values in Figs 8B, 9D and 9E. At each Δ*f*-value, durations integrated and segregated were extracted from 36 four minute simulations excluding the first and (incomplete) last duration. A global mean percept duration *T* ^glob^ was computed combining durations integrated and segregated across all simulations at each of the 8 Δ*f*-values used in the experiments. Durations were normalized by *T* ^glob^ before taking a mean to produce the normalized mean durations integrated *T* ^int^ and segregated *T* ^seg^ plotted in the figures.

### Comparison to standard statistical distributions

Note that the notations used to define these standard distributions are local to this section and not used elsewhere in the paper. The probability density function (pdf) for the gamma distribution is given by

$$f\left(x|k,\theta \right)=\frac{1}{\Gamma \left(k\right)\theta^{k}}x^{k-1}\exp\left(-x/\theta \right),$$ (15)

where *k* is the shape and *θ* the scale parameter. The mean of the distribution is equal to *k*/*θ* and the variance is equal to *k*/*θ* ^2^. The pdf for the log-normal distribution is given by

$$f\left(x|\mu ,\sigma \right)=\frac{1}{x\sigma \sqrt{2\pi }}\exp(-\frac{{\left(\ln x-\mu \right)}^{2}}{2\sigma^{2}})$$ (16)

where *μ* is the location parameter and *σ* the scale parameter. The mean of the distribution is equal to exp(*μ* + *σ* ^2^/2) and the variance is equal to (exp(*σ* ^2^) − 1) exp(2*μ* + *σ* ^2^).

In *Statistics of dominance durations: model*, the distributions from the model and from the experiment shown in Fig 5A and 5B were compared with the standard distributions given by Eqs (15) and (16) using a one-way Kolmogorov-Smirnov KS test. The null hypothesis is that the test data are drawn from the standard distribution and a significant result (*P* < 0.05) indicates that the test data are not drawn from the comparison distribution. In a further analysis, the model and experimental data were also compared in a two-way KS test to see if they come from the same distribution (without specifying what that distribution might be). The null hypothesis is that they have the same underlying distribution and a significant result (*P* < 0.05) indicates that they are drawn from different distributions.

### Repeated measures ANOVAs

Three subjects that did not contribute scores at all eight conditions (two at Δ*f* = 1 st, one at Δ*f* = 15 st) were excluded from the following analysis. A significance level of 0.05 is used throughout. In the text throughout the manuscript, the Greenhouse-Geisser (GG) corrected *P*-values are reported as appropriate where the data reached significant in a Mauchly sphericity test.

A one-way repeated measures ANOVA on proportion of time integrated (see Fig 9A) was performed with Δ*f* = {1, 2, 3, 5, 7, 9, 11, 15} as the within subjects factor. The analysis reported in Table 3 shows a significant effect of Δ*f* on proportion integrated *F*(7, 77) = 24.656, *P* < 0.001. Pairwise comparisons with Bonferroni-corrected significance levels showed that each individual condition has significant differences with at least two (and up to 5) other conditions.

**Table 3:** One-way repeated measures ANOVA of proportion integrated for the factor Δ*f* (eight conditions, see Fig 9A). Analysis shows a significant effect of Δ*f* on proportion integrated. Data for *N* = 12 subjects, see text. Mauchly test for sphericity reaches significance, Greenhouse-Geisser correct *P*-value reported in the text.

Source	Type III SS	df	MS	F	Sig.	G-G
DF	1.129	7	0.161	24.656	< 0.001	< 0.001
Error(DF)	0.504	77	0.007			

A two-way repeated measures ANOVA on log normalized durations $\log({\bar{T}}_{k}^{\text{int}}\left(j\right))$ and $\log({\bar{T}}_{k}^{\text{seg}}\left(j\right))$ (see Eqs (11) and (12)) was performed with Percept type (integrated or segregated) and frequency difference Δ*f* as within subjects factors. The use of a log transformation of the durations is standard for this type of experiment [19, 21]. The analysis reported in Table 4 shows a highly significant interaction for Percept * Δ*f*, *F*(7, 77) = 16.225, *P* < 0.001. As for the individual factors, Percept does not reach significance *F*(1, 11) = 3.158, *P* = 0.103, neither does Δ*f* *F*(7, 77) = 1.053, *P* = 0.402. We note that, given the interaction between Percept and Δ*f* it is hard to interpret effects of the individual factors. Pairwise comparisons with Bonferroni-corrected significance levels reveal no significant differences between individual conditions in this two-way analysis.

**Table 4:** Two-way repeated measures ANOVA of log noramlized durations for Percept type (integrated or segregated), Δ*f* (eight conditions) and their interaction, see Fig 9B. Analysis shows a significant interaction for Percept * Δ*f*. Data for *N* = 12 subjects, see text. Mauchly test for sphericity reaches significance for the Percept * Δ*f* interaction, Greenhouse-Geisser corrected *P*-value reported in the text.

Source	Type III SS	df	MS	F	Sig.	G-G
Percept	1.451	1	1.451	3.158	0.103	0.103
Error(Percept)	5.053	11	0.459			
Δ*f*	0.795	7	0.114	1.053	0.402	0.390
Error(Δ*f*)	8.304	77	0.108			
Percept * Δ*f*	7.603	7	1.086	16.225	< 0.001	< 0.001
Error(Percept * Δ*f*)	5.155	77	0.067			

A one-way repeated measures ANOVA on *η* with Δ*f* as a within subjects factor does not show a significant effect *F*(7, 77) = 0.878, *P* = 0.463, see Table 5.

**Table 5:** One-way repeated measures ANOVA for the measure *η* with Δ*f* as a factor (eight conditions) see Fig 9C. Analysis shows that effect of Δ*f* on *η* does not reach significance. Mauchly test for sphericity reaches significance, Greenhouse-Geisser corrected *P*-value reported in the text. Data for *N* = 12 subjects, see text.

Source	Type III SS	df	MS	F	Sig.	G-G
DF	5.715	7	0.816	0.878	0.528	0.463
Error(DF)	71.597	77	0.930			

## Supporting Information

S1 Data Percept durations from psychoacoustic experiments. Durations from each trial are provided in Matlab data format in the supporting information file S1_data.zip.
(ZIP)
